# *Agrobacterium tumefaciens*-mediated transformation and T-DNA insertion characterisation of *Didymella tanaceti* and *Stagonosporopsis tanaceti* for infection visualisation in pyrethrum

**DOI:** 10.1371/journal.pone.0355715

**Published:** 2026-08-10

**Authors:** Violeta Carrillo, Paul W. J. Taylor, Alexander Idnurm, Tamieka L. Pearce, Jason B. Scott, Niloofar Vaghefi

**Affiliations:** 1 School of Agriculture, Food and Ecosystem Sciences, University of Melbourne, Parkville, Victoria, Australia; 2 School of BioSciences, University of Melbourne, Parkville, Victoria, Australia; 3 Tasmanian Institute of Agriculture, University of Tasmania, Tasmania, Australia; 4 Centre for Crop Health, University of Southern Queensland, Queensland, Australia; Amity University, INDIA

## Abstract

*Agrobacterium tumefaciens*-mediated transformation (ATMT) has been widely used in filamentous fungi for gene functional studies and for the introduction of fluorescent markers to investigate infection biology. In pyrethrum (*Tanacetum cinerariifolium*), the foliar pathogens *Didymella tanaceti* and *Stagonosporopsis tanaceti* cause significant yield losses. However, the infection biology of *D. tanaceti* remains poorly understood. In this study, ATMT was used to generate fluorescently labelled strains of *D. tanaceti* and *S. tanaceti* expressing mNeonGreen and tdTomato, respectively. Transformants were characterised for fluorescence stability, morphology, growth, and pathogenicity. Whole-genome sequencing revealed predominantly single-copy T-DNA insertions, with occasional partial insertions and small genomic rearrangements. Despite variation in fluorescence intensity among *D. tanaceti* transformants, stable expression of fluorescent proteins was confirmed in both species. Detached leaf assays demonstrated that transformed strains exhibited infection patterns comparable to their respective wild types. In *D. tanaceti*, germination occurred by 24 hours after inoculation (HAI), followed by hyphal growth and direct epidermal penetration by 72 HAI, leading to mesophyll colonisation. No major differences in infection timing or colonisation patterns were observed between wild type and transformed strains. In *S. tanaceti*, infection dynamics were consistent with previous descriptions, supporting the suitability of transformed strains for *in planta* studies. Fluorescently labelled strains of *D. tanaceti* and *S. tanaceti a*re valuable tools for investigating infection biology and provide the first visual insights into early infection processes of *D. tanaceti* in pyrethrum. These findings lay the foundation for future studies incorporating quantitative approaches to better understand pathogen dynamics and interactions.

## Introduction

Pyrethrum (*Tanacetum cinerariifolium*) is a perennial crop in the Asteraceae family, cultivated as the primary source of pyrethrins, which are natural insecticidal compounds [1]. Pyrethrins are known for their ability to induce rapid insect knockdown whilst having low toxicity in mammals and rapid degradation in the environment [2]. Australia is currently the largest global producer, with most cultivation concentrated in Tasmania [3,4].

During its 18-month cultivation cycle between sowing and first harvest, pyrethrum is susceptible to several fungal pathogens. Currently, *Didymella tanaceti* is the most prevalent foliar fungal pathogen [5], followed by *Stagonosporopsis tanaceti* [6], both causing significant yield losses [7,8]. *Didymella tanaceti* causes the disease tan spot and *S. tanaceti* causes ray blight [5,9]. These ascomycetes primarily cause leaf lesions. Historically, *S. tanaceti* was the dominant pathogen of pyrethrum fields until 2009. Since then, *D. tanaceti* has become the most damaging spring pathogen, partly due to the evolution of resistance to succinate dehydrogenase inhibitor fungicide [10]. The biology and epidemiology of *S. tanaceti* are now well understood, enabling the development of targeted management strategies [9,11–13]. However, a significant knowledge gap remains regarding the infection biology of *D. tanaceti*. Understanding the infection process of *D. tanaceti* and its interaction with *S. tanaceti* is critical for developing specialised management strategies for tan spot.

Histological approaches have long been central to understanding plant-pathogen interactions, with staining techniques enabling the visualisation of pathogens *in planta* and the characterisation of infection structures. Traditional histopathology techniques provide key insights into how pathogens invade, colonise, and damage host tissues. However, they lack the specificity to rapidly distinguish individual species in mixed infections [14]. This limitation is particularly relevant given that plants in natural environments are rarely infected by a single pathogen species [15]. In contrast, fluorescently labelled pathogens provide a powerful means to track infection processes of specific pathogens *in planta* [14,16,17], enabling clear differentiation of the target pathogen from other microorganisms simultaneously colonising host tissues.

Since 1998, *Agrobacterium tumefaciens* mediated transformation (ATMT) has been extensively used in filamentous fungi to investigate gene function [18,19] and to insert fluorescent proteins, such as DsRed and EGFP, for studying plant-pathogen interactions [16,17,20,21]. The green fluorescent protein (GFP), derived from the jellyfish *Aequorea victoria*, is a highly stable reporter capable of resisting temperatures over 60 °C, low pH and proteases [20,22]. However, this wild type GFP is poorly translated in fungi, leading to the development of codon-optimised variants such as SGFP and EGFP, with SGFP preferred for filamentous fungi [20]. Over 10 years ago, the discovery of the yellow fluorescent protein LanYFP from the lancelet *Branchiostoma lanceolatum* led to the engineering of mNeonGreen, a monomeric, bright green fluorescent protein [23] that has been expressed in filamentous fungi such as *Aspergillus fumigatus* [24] and *Sordaria macrospora* [25]. Similarly, the discovery of red fluorescent proteins expanded the available spectral range for live imaging. The original red fluorescent protein, DsRed from *Discosoma* spp., was later engineered into monomeric and dimeric variants with enhanced brightness and photostability, such as mRFP1 and tdTomato [26,27]. The latter has been successfully incorporated into *Sclerotium rolfsii* [28] and *Aspergillus niger* [29], producing a characteristic bright red-yellow fluorescence.

This study aimed to transform *D. tanaceti* and *S. tanaceti* via ATMT to express mNeonGreen and tdTomato, respectively. The infection capacity and transformant stability of selected transformed strains were also corroborated. These fluorescently labelled variants enabled the detailed visualisation of fungal infection within pyrethrum leaves. Hence, this report provides a first look into the early stages of the infection process of *D. tanaceti* and demonstrates the utility of fluorescently labelling as a powerful tool to distinguish fungal structures within plant tissue.

## Materials and methods

### Fungal cultures and spore suspension preparation

Strain BRIP 61988 [30] was selected as the representative wild type of *D. tanaceti*, and isolate UOM ST2 was selected as the representative isolate of *S. tanaceti*. Pure cultures of each species were obtained by single spore isolation. Briefly, upon sporulation, 5 ml of autoclaved reverse osmosis (RO) water was added to each plate, and the surface was scraped with a sterile scalpel blade. The resulting spore suspensions were filtered using an autoclaved funnel and a single layer of Miracloth® (Millipore, Germany; pore size: 22–25 µm). Spore suspensions of each species were adjusted to 1 × 10^5^ spores/ml with a hemocytometer (Livingston, Australia) and spread across a Petri plate containing water agar (WA) (S1 Protocol). Under the dissecting microscope (Leica Microsystems, Germany), single spores were picked up and transferred to different media. That is, *D*. *tanaceti* pure cultures were grown on potato dextrose agar (PDA; Difco™, BD, Sparks, MD, USA), and *S. tanaceti* pure cultures were grown on V8 juice agar (V8; Campbell Soup Company, Lemnos, VIC, Australia) [13] (S1 Protocol). All cultures were kept at 21°C at a 12-hour light and dark cycle in a controlled growth chamber (MIR-254-PE, PHCBI, Japan) for 20 days.

### *Agrobacterium*-mediated transformation

#### Plasmids.

Targeted isolates of *D. tanaceti* and *S. tanaceti* were transformed with T-DNAs from the binary vectors pMAI31 (11,127 bp) and pMAI32 (11,847 bp), respectively. Both plasmids were constructed by modifying the T-DNA section of pLAU2 [31,32] that contains the kanamycin resistance gene *kanR*. Both plasmids carried the *kanR* gene and the fluorescent protein genes: pMAI31 (S1 Fig) contains *mNeonGreen* and pMAI32 (S2 Fig) contains *tdTomato*, each driven by the *act1* promoter and *trp3* terminator from *Leptosphaeria maculans*. The hygromycin resistance gene *hygR* was driven by *trpC* promoter and terminator from *Aspergillus nidulans.*

### Co-culture of *Agrobacterium* and fungi, and initial antibiotic selection

For the fungal transformation, spore suspensions from *D. tanaceti* and *S. tanaceti* wild type strains were obtained by adding 5 ml of autoclaved RO water to each plate. The surface was scraped with a sterile scalpel blade, and the resulting spore suspensions were filtered using an autoclaved funnel and a single layer of Miracloth®. The spore suspension obtained from each strain was adjusted to a concentration of 2–3 × 10^7^ spores/ml.

Liquid cultures of *Agrobacterium* strain EHA105 containing the plasmids were diluted with Lysogeny Broth (LB; 10 g tryptone, 5 g yeast extract, 10 g NaCl L ⁻ ^1^) [33] to an optical density at 600nm (OD_600_) of 0.6. In a 2 ml tube, 400 μl of diluted *Agrobacterium* was mixed with 400 μl of fungal spore suspension. From the admixture, 400 μl was transferred to a 15 cm Petri plate containing induction media (IM) consisting of 2.5 × MM salts (KH_2_PO_4_, K_2_HPO_4_, NaCl, MgSO_4_·7H_2_O, CaCl_2_·2H_2_O, FeSO_4_·7H_2_O and (NH_4_)_2_SO_4_), supplemented with glucose (0.6 g L ⁻ ^1^), glycerol (5 mL L ⁻ ^1^), MES buffer (50 mM, pH 5.3), agar (20 g L ⁻ ^1^) and acetosyringone (200 µM). The complete formulation is provided in Supplementary Protocol S2. Plates were incubated at 22°C in darkness for 2–3 days. *Agrobacterium tumefaciens* harbouring plasmid pMAI31 was co-cultured with spore suspension of strain BRIP 61988 (*D. tanaceti*). Separately, *A. tumefaciens* containing pMAI32 was co-cultured with the spore suspension of strain UOM ST2 (*S. tanaceti*).

In a biosafety cabinet, 25 ml overlay media (PDA for *D. tanaceti* and V8 for *S. tanaceti*) with 100 µg/ml cefotaxime (Fluorochem, United Kingdom) and 50 µg/ml hygromycin (Thermo Fisher Scientific, USA) was poured as an overlay and left to dry for 10 minutes. Plates were then sealed with parafilm and incubated at 22°C in darkness. Colony formation was monitored daily for 10 days. Colonies were transferred onto fresh PDA or V8 plates containing hygromycin and cefotaxime for an additional selection round.

### Establishment of pure cultures and stability of transformants

*Didymella tanaceti* transformants were single-spored to establish pure cultures of the transformed strains. Spore suspensions of *D. tanaceti* transformants were prepared from 3-week-old transformed plates, adjusted to 1 × 10^5^ spores/ml by counting the spores on a hemocytometer and diluting the suspension with RO water. Then, 100 μl of spore suspension was placed on a Petri plate containing WA, and the content was spread using a sterile rod. After 24 hours, germinating spores were observed under the dissecting microscope, picked up with a sterile needle, transferred onto PDA and kept at 22°C at a 12-hour light and dark cycle. *Stagonosporopsis tanaceti* transformants were hyphal-tipped by sampling single hypha from the margin of one-week-old cultures and transferring to V8 agar, as this species did not consistently produce spores under the conditions used. Cultures were kept at 22°C at a 12-hour light and dark cycle.

Two consecutive rounds of culturing on PDA and V8 agar media (for *D. tanaceti* and *S. tanaceti* transformant, respectively) containing hygromycin and cefotaxime were performed before subculturing in antibiotic-free media.

### Fluorescence microscopy and quantification

The fluorescence of spores and hyphae samples, obtained from pure cultures of transformed strains, was observed by mounting a 5 μl spore suspension droplet and 2–3 mm^2^ agar plug on a glass slide. Fluorescence was confirmed using compound microscope Leica DM6000 (Leica Microsystems, Germany). To confirm the specificity of fluorescence signals samples were observed using the Yellow Fluorescent Protein filter cube (YFP, excitation: 490–510 nm, dichroic mirror 515 nm, emission: 520–550 nm band-pass) to detect mNeonGreen and the DsRed fluorescent filter cube (excitation: 540–552 nm, dichroic mirror 560 nm, emission: 567–643 nm band-pass) to detect tdTomato.

Hyphae fluorescence intensity, obtained from pure cultures of transformed strains, was quantified using Fiji image processing software version 2.16.0/1.54p [34], and splitting the channels to measure the fluorescence in grey-scale values. Fifteen regions of interest (ROIs; 1.96 × 1.96 µm square) were analysed per transformant from multiple hyphal regions within a single biological replicate per transformed strain. Regions of interest were placed over fluorescent hyphal regions and background intensity was measured from adjacent non-fluorescent areas of equal size. The background noise was subtracted to obtain the fluorescence intensity values, which were then used to calculate the corrected total cell fluorescence (CTCF) value [35,36].


$$\begin{array}{c} \text{CTCF}=\text{Integrated density}-(\text{Area of selected cell x Mean fluorescence of background reading}) \end{array}$$

Images were captured at a 40 × magnification with 1 s exposure using the Leica Application Suite (LAS) Software. One-way ANOVA and Tukey post hoc test were used to compare fluorescence between samples. Based on CTCF values, *D. tanaceti* transformants UOM DT1 and UOM DT5, and *S. tanaceti* transformants UOM STC1R2 and UOM STC4R2, were selected for molecular characterisation.

### Growth rate and morphological characterisation

Growth rates were measured in triplicate for each strain on PDA (*D. tanaceti*) and V8 agar (*S. tanaceti*). A single 5 mm diameter agar plug was placed at the centre of a marked 90 mm Petri plate. All cultures were kept at 22°C at 12-hour light/dark cycle. Colony diameter was measured along a single axis passing through the centre of the plate. As colonies exhibited regular, circular growth with smooth margins, a single measurement was considered sufficient to estimate colony size. Days until sporulation were recorded for each strain, defined as the time (in days) from inoculation to the first visible production of conidia.

Spore shape and size were assessed by measuring the length and width of 35 spores from each of the five *D. tanaceti* transformants. In addition, conidial dimensions were measured for the wild type *S. tanaceti* isolate UOM ST2 and the transformed isolate UOM STC1R2, which were sporulating at the time of sampling. Spore suspensions were prepared from two-week-old cultures kept at 22°C at 12-hour light/dark cycle, by placing 5 ml of autoclaved RO water and scraping the agar surface with a sterile blade. The liquid was then filtered using an autoclaved funnel wrapped with Miracloth into 50-ml centrifuge tubes. Spore suspensions were mounted on glass slides and examined under a compound microscope (Leica DM6000) at 100 × magnification. Measurements were taken using the analysis tools available in the LAS software.

### Statistical analyses

Statistical analyses were conducted in R (version 4.5.0) [37]. An alpha level of 0.05 was used. Data were assessed for normality and homogeneity of variance through visual inspection of residual plots, Shapiro-Wilk tests, and Levene’s test. Fluorescence intensity (CTCF), conidial dimensions (length and width) and growth data were analysed using one-way ANOVA. For growth measurements, analyses were performed separately at each time point to compare differences among strains. When significant effects were detected, pairwise comparisons were conducted using estimated marginal means (emmeans) with Tukey adjustment [38].

### Molecular characterisation of *S. tanaceti* and *D. tanaceti* transformants

Two transformed strains from each species were selected for whole-genome sequencing to determine the copy number of T-DNA insertions and their locations in the genomes. For *D. tanaceti*, transformed strains UOM DT1 and UOM DT5, and BRIP 61988 wild type were selected, and for *S. tanaceti*, strains UOM STC1R2 and UOM ST4R2 were chosen for genome sequencing.

Subcultures were grown in potato dextrose broth (PDB, Difco, Australia) in a shaker at 25°C for two weeks. In the biosafety cabinet, mycelium samples were collected with sterilised tweezers and placed in an autoclaved paper towel (autoclave cycle: 121°C for 20 min) to remove the excess liquid. The mycelia were transferred to 2 ml safe-lock tubes and placed immediately in a cooler containing liquid nitrogen. Samples were freeze-dried for 48 hours in a freeze dryer (Biobase). Two magnetic beads were then added to each tube, and samples were lysed for 30 s at 30 Hz frequency in the TissueLyser III (Qiagen). DNA was extracted following the manufacturer’s instructions for the DNeasy Plant Mini Kit (Qiagen, Germany). DNA quality was assessed via gel electrophoresis (1.5% agarose) and concentration was measured using the Qubit 2.0 fluorometer (Thermo Fisher Scientific).

Whole genome sequencing of the selected strains was performed using the Illumina Sequencing platform at the Australian Genome Research Facility (AGRF, Melbourne) as paired-end reads of 150 bp. The NovaSeq Control Software (NCS) v1.3.0.39308 and Real-Time Analysis (RTA) v4.29.2 performed image analysis in real-time. Illumina DRAGEN BCL Convert 07.031.732.4.3.6 pipeline was used to generate the sequence data. Adapter sequences were removed with Trimmomatic (Galaxy Version 0.36.6) and the genome was assembled using SPAdes (Galaxy Version 4.1.0 + galaxy0) [39]. Assembly quality and completeness were assessed using Quast (Galaxy version 5.3.0 + galaxy0) and BUSCO (Galaxy version 5.8.0 + galaxy1) using the *Pleosporales* lineage dataset (pleosporales_odb10; n = 6,641 orthologous groups) in BUSCO v5 lineage datasets (odb10). The Kmer counting software Jellyfish v.2.3.1 [40] was used to estimate the genome size using raw Illumina data.

The number of T-DNA inserts of each strain was determined by custom BLAST in Geneious Prime v2025.0.3 (https://www.geneious.com), using the T-DNA sequence as query against the assembled genomes of the transformed strains. Genes were predicted using AUGUSTUS [39] with *Botrytis cinerea* as the reference species. The predicted gene models were subjected to BLASTx searches against protein databases to infer putative gene functions. Insertion sites were identified by mapping the 150 bp paired reads against the T-DNA of each strain and BLAST analysis using the flanking regions as queries against the annotated wild type genomes. Gene insertion sites were illustrated schematically using BioRender (https://biorender.com) to visualise the T-DNA integration and associated genomic features.

### Detached leaves assay of wild type and transformed strains

To confirm infection and assess whether transformed strains showed infection behaviour comparable to that of the corresponding wild type strains, detached leaf assays were conducted using pyrethrum leaflets obtained from 7-month-old pyrethrum plants grown under controlled conditions (20°C, 10 h light/14 h dark, 60% relative humidity). Leaflets were inoculated with spore suspensions (1 × 10⁷ spores/ml) supplemented with Tween 20 (0.02% v/v). A 5–10 µl droplet was placed at the centre of each leaflet and spread across the surface using a pipette tip. Control leaflets received RO water containing Tween 20 (0.02% v/v). After inoculation, samples were placed in glass Petri plates containing moistened sterile paper towels to maintain humidity and incubated at 22°C under a 12 h light/12 h dark cycle. Each leaflet was considered an independent biological replicate.

For *D. tanaceti*, a total of 46 leaflets were used, with 23 inoculated with the wild type strain (BRIP 61988) and 23 with the transformed strain (UOM DT5). Five leaflets per treatment were sampled at 24, 48, 72, and 120 hours after inoculation (HAI) to assess germination and early infection. At 24 and 48 HAI, leaflets were observed from the adaxial surface using a compound microscope (Leica DM6000). At 72 and 120 HAI, samples were transversely sectioned by hand using a razor blade, with tissues stabilised between polystyrene sheets. Wild type-infected tissues were cleared in a 1:1 (v/v) ethanol:glacial acetic acid solution for 12 h and stained with lactophenol cotton blue prior to observation under brightfield microscopy. Fluorescent transformants were examined directly using the YFP filter set.

For *S. tanaceti*, ten leaflets were used, with five inoculated with the wild type strain (UOM ST2) and five with the transformed strain (UOM STC1R2). Leaflets were sampled at 72 HAI and transversely sectioned using a dissecting microscope. Sections were observed under a compound microscope equipped with a DsRed filter to visualise fungal colonisation *in planta*. Transformants were considered phenotypically similar to the wild type if symptom development timing and colonisation patterns were comparable to the literature on *S. tanaceti* [41]. Each leaflet was considered an independent biological replicate. No technical replicates were performed [41].

## Results

### *Agrobacterium*-mediated transformation of *D. tanaceti* and *S. tanaceti*

Following the co-culture of *Agrobacterium* harbouring the plasmids and the spore suspensions of the wild type isolate BRIP 61988 and UOM ST2, five hygromycin-resistant colonies of *D. tanaceti* UOM DT1, UOM DT2, UOM DT3, UOM DT4 and UOM DT5, and four of *S. tanaceti* UOM STC1R2, UOM STC2R2, UOM STC3R2, and UOM STC4R2 were recovered. All transformant strains of both species consistently retained fluorescence after repeated subculturing, indicating successful and stable retention of the construct.

### Fluorescence microscopy and CTCF values

**Fig 1:**
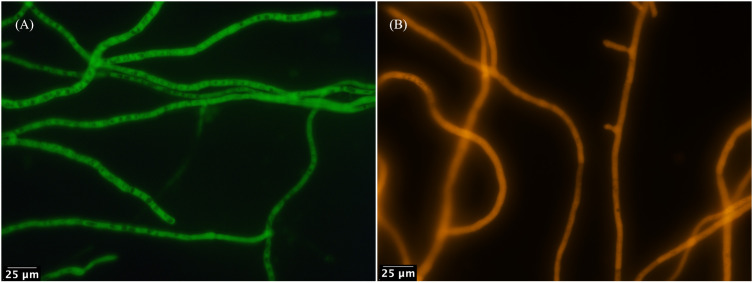
Fluorescent microscopy images of transformed *Didymella tanaceti* and *Stagonosporopsis tanaceti.* (A) Mycelium of strain UOM DT5 observed under a YFP filter (excitation 513 nm, emission 527 nm) showing expression of the mNeonGreen fluorescent protein. (B) Mycelium of strain UOM STC1R2 observed under a DsRed filter (excitation 558 nm, emission 583 nm) showing expression of the tdTomato fluorescent protein. Scale bar = 25 μm.

Fluorescence in both species was confirmed under the compound microscope, using the YFP filter to observe *D. tanaceti* strains containing the mNeonGreen fluorescent protein, and the DsRed filter to observe the *S. tanaceti* strains containing tdTomato fluorescent protein. Spores and hyphae of both species exhibited strong fluorescence. *Didymella tanaceti* transformant strains expressing mNeonGreen displayed green fluorescence (Fig 1 A) with variable intensities among strains, while the *S. tanaceti* transformant strains expressing tdTomato exhibited red-orange fluorescence (Fig 1 B) with no apparent qualitative differences between strains. Fluorescence was strain-specific, with no signal detected outside the corresponding channels. *Didymella tanaceti* transformants expressing mNeonGreen did not emit fluorescence in the DsRed channel, and *S. tanaceti* transformants expressing tdTomato showed no fluorescence in the green channel.

**Fig 2:**
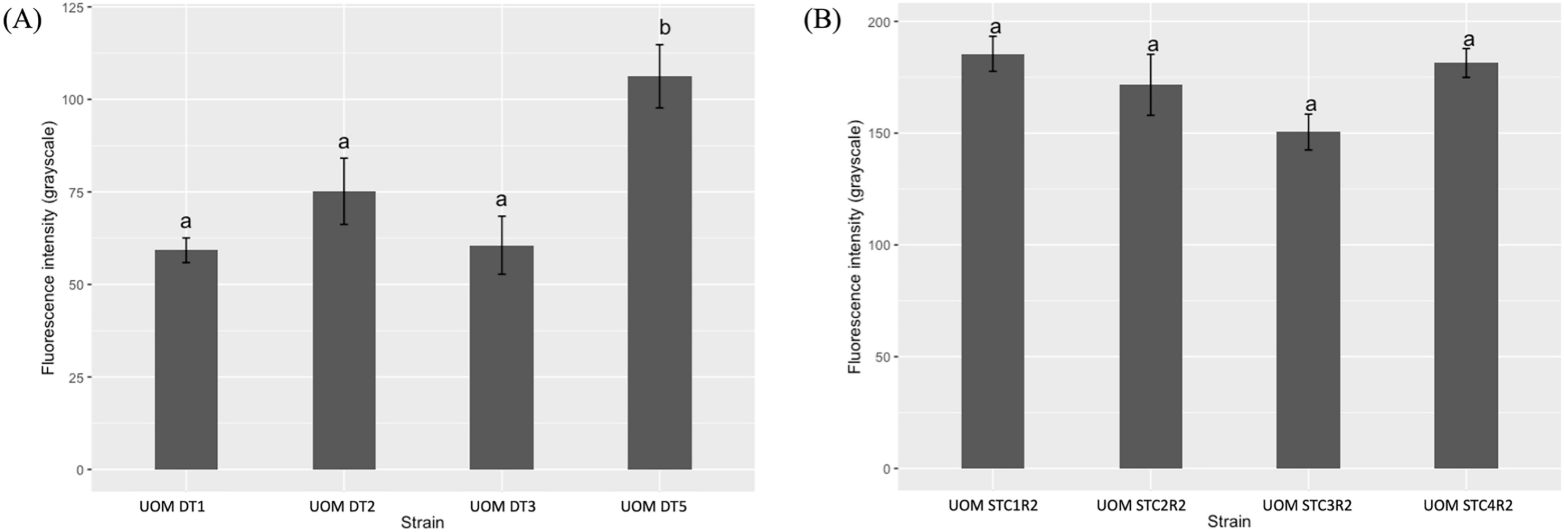
Fluorescence intensity of transformed strains of *Didymella tanaceti* and *Stagonosporopsis tanaceti* based on greyscale-derived corrected total cell fluorescence (CTCF) values. Bars represent mean ± standard error (SE). Different letters indicate statistically significant differences among strains based on estimated marginal means (emmeans) with Tukey adjustment (α = 0.05). (A) *Didymella tanaceti*: strain UOM DT5 exhibited significantly higher fluorescence intensity compared with the other strains. (B) *Stagonosporopsis tanaceti*: no significant differences in fluorescence intensity were detected among transformed strains.

No apparent differences in fluorescence intensity were observed among the four *S. tanaceti* transformant strains. This was supported by statistical analysis, which showed no significant differences in CTCF values among strains (one-way ANOVA, *P* = 0.0504; Fig 2B). In *D. tanaceti*, however, a significant effect of strain was detected (one-way ANOVA, *P* = 9.982e-05; Fig 2A). Strain UOM DT5 exhibited significantly higher CTCF values compared with strains UOM DT1 (*P* = 0.0003), UOM DT2 (*P* = 0.0249) and UOM DT3 (*P* = 0.0004).

### Morphological comparison against the wild type

Colony morphology remained the same across cultures. *D. tanaceti* wild type and transformed strains exhibited the characteristic green olivaceous mycelia (S3 Fig), with sporulation observed after seven days. Likewise, *S. tanaceti* wild type and transformants developed white mycelia during the first week (S4 Fig), with pycnidia appearing three weeks post-subculturing in wild type strain UOM ST2 and transformed strain UOM STC1R2. Colony growth rates of *S. tanaceti* were consistent across all strains, with complete coverage of 90 mm Petri plates achieved within nine days (S1 Table). In contrast, one *D. tanaceti* transformed strain (UOM DT4) displayed significantly reduced growth rate compared to the wild type (p = 0.008) and was therefore excluded from subsequent analyses (S2 Table).

Spore shape of *D. tanaceti* transformed isolates was cylindrical to ellipsoidal with rounded ends, rarely septate. Spores of the wild type isolate BRIP 61988 measured 4.6–6.8 × 2.4–3.4 µm. For UOM DT1, spores measured 4.3–7.1 × 2.6–4.2 µm; UOM DT2, 5.3–7.6 × 2.7–4.7 µm; UOM DT3, 4.3–7.3 × 2.5–4.0 µm; UOM DT4, 5.0–8.0 × 1.3–4.7 µm; and UOM DT5, 5.9–8.5 × 2.9–4.7 µm.

Spores of *S. tanaceti* wild type and transformed isolates were hyaline, aseptate, and ellipsoidal. Spores of the wild type isolate UOM ST2 measured 4.6–6.4 × 1.9–3.1 µm, whereas the spores of the transformed isolate UOM STC1R2 measured 5.1–7.6 × 1.9–3.0 µm.

Spore length (F_5,204_ = 21.95, P < 0.001) and width (F_5,204_ = 9.94, P < 0.001) differed significantly among *D. tanaceti* isolates (Table 1). For *S. tanaceti*, spore length differed significantly between the wild type and transformed isolate UOM STC1R2 (F_1,68_ = 17.83, P < 0.001), whereas spore width did not (F₁,₆₈ = 0.82, P = 0.368).

**Table 1:** Conidial length and width of wild type and transformed isolates of *Didymella tanaceti* and *Stagonosporopsis tanaceti.*

Species	Isolate	Length (µm)	Width (µm)
*D. tanaceti*	BRIP 61988	5.58 ± 0.11 a	3.02 ± 0.07 a
*D. tanaceti*	UOM DT1	5.78 ± 0.11 a	3.24 ± 0.07 ab
*D. tanaceti*	UOM DT3	6.02 ± 0.11 ab	3.27 ± 0.07 ab
*D. tanaceti*	UOM DT2	6.39 ± 0.11 bc	3.63 ± 0.07 c
*D. tanaceti*	UOM DT4	6.58 ± 0.11 cd	3.48 ± 0.07 bc
*D. tanaceti*	UOM DT5	6.99 ± 0.11 d	3.59 ± 0.07 c
*S. tanaceti*	UOM ST2	5.54 ± 0.08 a	2.49 ± 0.05 a
*S. tanaceti*	UOM STC1R2	6.01 ± 0.08 b	2.55 ± 0.05 a

**Note**: Values represent estimated marginal means ± SE. Means followed by the same letter within each species and column are not significantly different according to pairwise comparisons of estimated marginal means (P < 0.05).

### Molecular characterisation of T-DNA insertion sites

The assembly size of the wild type *D. tanaceti* strain BRIP 61988 was 41.1 Mbp, with a sequencing coverage of 141 × , considering contigs >1,000 bp in length. The transformed strains UOM DT1 and UOM DT5 had an assembly size of 39.4 Mbp and 38.9 Mbp, with sequencing coverages of 205× and 160 × , respectively. The estimated genome size for *D. tanaceti* isolates was 40.7 Mbp using Jellyfish v.2.3.1. For *S. tanaceti* transformed strains UOM STC1R2 and UOM STC4R2, the assembly sizes were 33.3 Mbp and 33.4 Mbp, respectively, considering contigs >1,000 bp in length, and the sequencing coverages were 68.6× and 73.5 × , respectively. The estimated genome size for *S. tanaceti* was 35.5 Mbp. Assembly statistics, including contig counts and N50 values, are summarised in Table 2.

**Table 2:** Assembly statistics and genome size estimates for wild type and transformed isolates of *Didymella tanaceti* and *Stagonosporopsis tanaceti.*

Species	Strain	Assembly size (Mbp)	Sequencing coverage (×)	Genome size estimate (Mbp)	N° of contigs	N50 (bp)	GC (%)	Notes
*D. tanaceti*	BRIP 61988 (wild type)	41.1	141	40.7	744	184976	52.94	Contigs >1,000 bp
*D. tanaceti*	UOM DT1	39.4	205	40.7	658	188218	53.05	Contigs >1,000 bp
*D. tanaceti*	UOM DT5	38.9	160	40.7	718	191554	53.05	Contigs >1,000 bp
*S. tanaceti*	UOM STC1R2	33.3	68.6	35.5	150	470240	52.66	Contigs >1,000 bp
*S. tanaceti*	UOM STC4R2	33.4	73.5	35.5	126	565383	52.66	Contigs >1,000 bp

**Note:** Genome size estimates were obtained using Jellyfish v.2.3.1. Assembly statistics were calculated using contigs >1,000 bp in length.

BUSCO analysis against 6,641 orthologous groups indicated high completeness for the *D. tanaceti* wild type and transformed strains. The wild type strain BRIP 61988 recovered 95.5% of expected genes (6,341 complete BUSCOs), of which 95.2% (6,325) were single-copy and 0.2% (16) were duplicated, while 1.3% (84) were fragmented and 3.3% (216) were missing. A similar completeness was observed in the transformed strains UOM DT1 and UOM DT5. The former displayed 95.5% completeness (6,341 complete BUSCOs), with 95.2% (6,325) single-copy, 0.2% (16) duplicated, 1.3% (84) fragmented, and 3.3% (216) missing. The latter showed a nearly identical profile, with 95.6% of genes detected (6,346 complete BUSCOs), comprising 95.3% (6,330) single-copy and 0.2% (16) duplicated, alongside 1.2% (78) fragmented and 3.3% (217) missing.

For *S. tanaceti* transformed strains, BUSCO analysis against 6,641 orthologous groups indicated high completeness. Strain UOM STC1R2 had 96.1% of expected genes detected (6,381 complete BUSCOs). Of these, 95.9% (6,370) were present as single-copy and 0.2% (11) as duplicated, while 1.2% (78) were fragmented and 2.7% (182) were missing. Similarly, for strain UOM STC4R2, 96.0% of genes were recovered (6,374 complete BUSCOs), comprising 95.8% (6,364) single-copy and 0.2% (10) duplicated, with 1.2% (80) fragmented and 2.8% (187) missing.

The T-DNA construct in *D. tanaceti* transformants carrying the mNeonGreen fluorescent protein gene was 4,951 bp in length, while the T-DNA construct in *S. tanaceti* transformant carrying the tdTomato fluorescent protein gene was 5,671 bp. BLASTn searches of the T-DNA sequence against each transformed genome assembly identified contigs containing the T-DNA. Strains UOM DT1, UOM STC1R2, and UOM STC4R2 each contained a single T-DNA insertion. Strain UOM DT5, however, carried a single T-DNA insertion as well as an additional partial insertion of 957 bp corresponding to the *trp3* terminator located on a different contig. The sizes of the T-DNA insertions were 4,940 bp in UOM DT1 and 3,972 bp in UOM DT5, whereas the insertions in UOM STC1R2 and UOM STC4R2 measured 5,631 bp and 5,662 bp, respectively.

**Fig 3:**
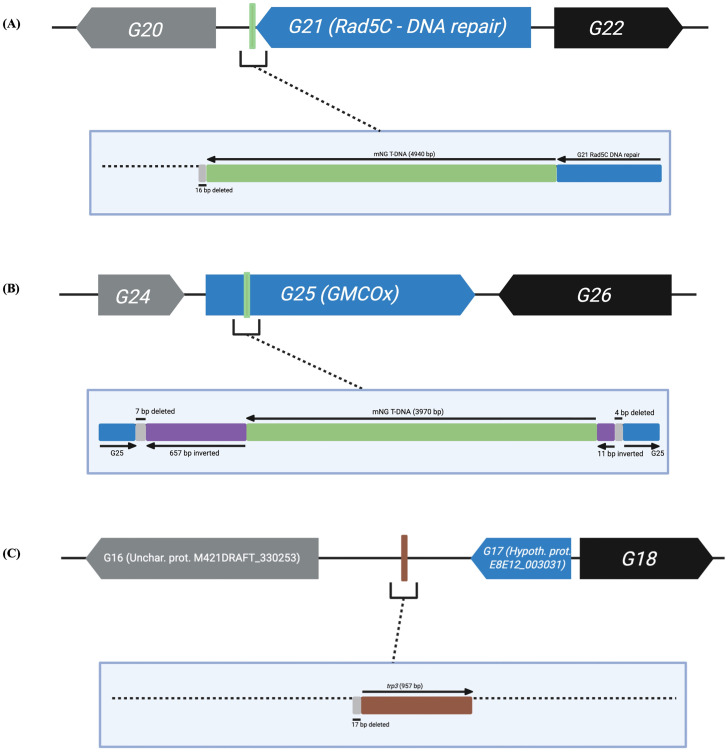
T-DNA insertion sites in *Didymella tanaceti* transformants. (A) Schematic representation of the T-DNA insertion site (green line) in strain UOM DT1. Alignment against the wild type showed a 16 bp deletion at the insertion site. (B) Schematic representation of the T-DNA insertion site (green line) in strain UOM DT5. Alignment with the wild type revealed an 7 bp deletion and a 657 bp inversion downstream of the insertion site, as well as an 11 bp inversion and a 4 bp deletion upstream of the insertion site. (C) Schematic representation of a partial T-DNA insertion in strain UOM DT5, comprising 957 bp of the trp3 terminator. Alignment against the wild type showed a 17 bp deletion at insertion site.

The specific genomic position of the T-DNA insertion in *D. tanaceti* UOM DT1, was identified downstream of gene G21 (Fig 3A), which showed the highest similarity to a DNA repair and recombination protein rad5C from *Stagonosporopsis vannaccii* (NCBI accession: KAJ4988673.1; query coverage: 84%; per. identity: 68.91%). Alignment with the wild type revealed a 16 bp deletion at the insertion site (Fig 3B). In *D. tanaceti* UOM DT5, the complete T-DNA was inserted within gene G25 (Fig 3C), which showed highest similarity to a GMC oxidoreductase (GMCox) from *S.vannaccii* (accession: KAJ4983148.1; query cover: 93%; per. identity: 77.61%). A comparison with the wild type identified a seven bp deletion and a 657 bp inversion downstream the T-DNA, and an 11 bp inversion and four bp deletion upstream of the T-DNA, within GMCox (Fig 3B). Additionally, a 957 bp region corresponding to the T-DNA terminator was located between genes G16 and G17 (Fig 3C). BLAST results indicate that G16 shared the highest similarity with an uncharacterised protein (M421DRAFT_330253) from *Didymella exigua* (accession number: XP_033443501.1; query coverage: 49%; percentage identity: 82.45%). On the other hand, G17 showed highest similarity to a hypothetical protein (E8E12_003031) from *Didymella heteroderae* (accession number: KAF3039328.1; query coverage: 82%; percentage identity: 53.11%)

**Fig 4:**
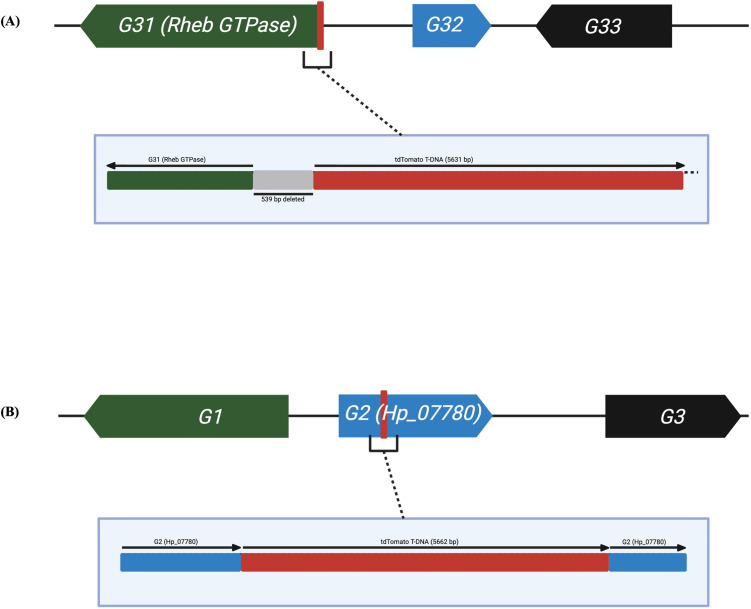
T-DNA insertion sites in *Stagonosporopsis tanaceti* transformants. (A) Schematic of the T-DNA insertion site (red line) in strain UOM STC1R2. Alignment with the wild type revealed a 539 bp deletion at the insertion site. (B) Schematic of the T-DNA insertion site (red line) in strain UOM STC4R2. Alignment with the wild type showed no deletions at the insertion site.

In *S. tanaceti* UOM STC1R2, the T-DNA was inserted within gene G31 (Fig 4A), which showed the highest similarity to a Rheb small monomeric GTPase from *S. vannaccii* (NCBI accession: KAJ4987483.1; query coverage: 100%; per identity: 75.32%). A 539 bp deletion was present at the insertion site. In *S. tanaceti* UOM STC4R2, the T-DNA was inserted downstream within gene G2 (Fig 4B), which showed similarity to a hypothetical protein (SVAN01_07780) from *S. vannaccii* (accession: KAJ4986721.1; query coverage: 31%; per. identity: 88.73%). No deletions were detected at this insertion site.

A summary of T-DNA insertion sites for all strains and associated genomic features is provided in Table 3.

**Table 3:** Summary of T-DNA insertion sites and associated genomic features in transformed strains.

Strain	Gene ID	Location of insertion	Insertion type	BLAST top hit	Species	Identity (%)	Query coverage (%)	Notes
**UOM DT1**	G21	downstream	complete	Rad5C DNA repair protein	*Stagonosporopsis vannaccii*	68.91	84	16 bp deletion at insertion site
**UOM DT5**	G25	within gene	complete	GMC oxidoreductase	*S. vannaccii*	77.61	93	deletions + inversions
UOM DT5*	G16	nearby gene	partial	uncharacterised	*Didymella exigua*	82.45	49	flanking region
UOM DT5*	G17	nearby gene	partial	hypothetical protein	*D. heteroderae*	53.11	82	flanking region
**UOM STC1R2**	G31	within gene	complete	Rheb GTPase	*S. vannaccii*	75.32	100	539 bp deletions

### Comparisons of wild types vs transformed strains during plant disease

**Fig 5:**
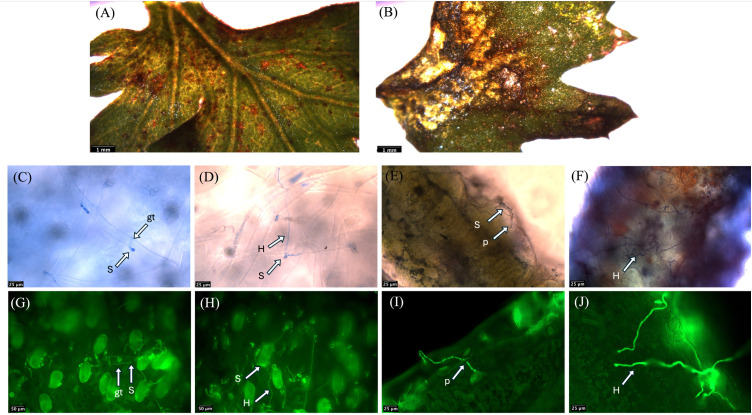
Tan spot lesions on pyrethrum leaves caused by *Didymella tanaceti* wild type and transformed strains, and subsequent disease progression. (A) Lesions caused by the non-transformed strain BRIP 61988 at 7 days after inoculation (DAI). (B) Lesions caused by the transformed strain UOM DT5 at 7 DAI. (C–F) Disease progression of the wild type isolate at 24, 48, 72, and 120 hours after inoculation (HAI), respectively. (G–J) Disease progression of the transformed strain UOM DT5 at the same time points. Panels C, D, G, and H show the adaxial surface of infected leaves, while panels E, F, I, and J show transverse sections. Germ tubes were observed emerging from spores and directly penetrating the epidermis, followed by hyphal colonisation of the mesophyll. “S” indicates spore; “gt” indicates germ tube; “H” indicates fungal hyphae; “P” indicates penetration point; white arrows indicate fungal structures and sites of penetration. Scale bars: A–B = 1 mm; C–J = 25–50 μm.

Initial symptoms were observed 120 HAI for both the *D. tanaceti* wild type strain and the transformed strain UOM DT5, appearing as dark brown necrotic lesions on leaf surfaces and margins. Not all inoculated leaves developed visible symptoms. At seven days after inoculation (DAI), necrotic lesions on symptomatic leaves had enlarged and, in some cases, coalesced (Fig 5A,B).

Spore germination was detected 24 hours after inoculation (HAI) in both wild type and transformed strains, based on observations across replicated leaf samples (n = 5 per time point). In the wild type, spores typically formed swollen short chains before germ tube emergence (Fig 5C), a feature also observed in the transformed strain UOM DT5 (Fig 5G). At 48 HAI, both strains produced hyphae extending across the leaf surface, without evidence of specialised infection structures (Fig 5D, H). By 72 HAI, direct penetration through the epidermis into the mesophyll was observed (Fig 5E, I), and by 120 HAI, both strains had colonised the mesophyll through the middle lamella (Fig 5F, J). No differences in germination pattern, penetration timing, or tissue colonisation were observed between wild type and transformed strains.

**Fig 6:**
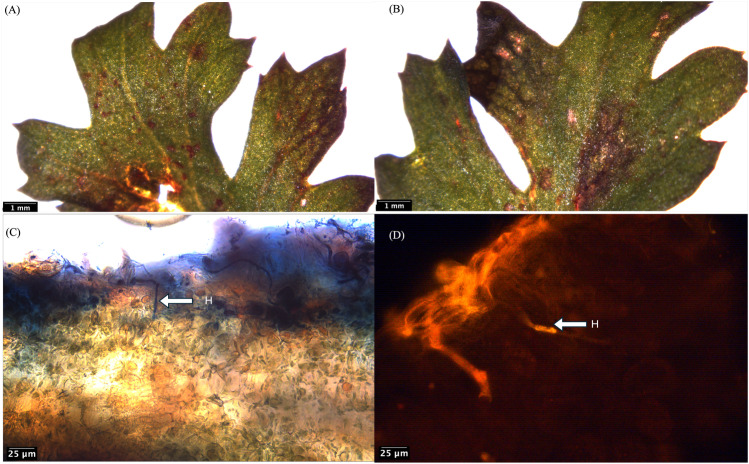
Lesions and tissue colonisation of pyrethrum leaves caused by *Stagonosporopsis tanaceti* wild type and transformed strains. (A) Lesions caused by the wild type strain at 7 days after inoculation (DAI). (B) Lesions caused by the transformed strain UOM STC1R2 at 7 DAI. (C) Transverse section of leaf tissue infected with the transformed strain UOM STC1R2 at 72 hours after inoculation (HAI). (D) Transverse section of leaf tissue infected with the wild type strain at 72 HAI. Fungal hyphae in the mesophyll are indicated by “H” and white arrows. Scale bars: A–B = 1 mm; C–D = 25 μm.

For *S. tanaceti*, initial symptoms appeared at 4 DAI as dark necrotic lesions at the leaf margins. Symptomatic leaves were transversally sectioned at approximately 50–100 µm and observed under a compound microscope using the DsRed fluorescent filter at 200× and 400 × magnification. The characteristic red fluorescence of UOM STC1R2 was detected in mycelium infecting the mesophyll at 72 HAI (Fig 6A). By 7 DAI, necrotic lesions extended across the entire or partial section of the leaf surface, irrespective of the strain (Fig 6A, B).

## Discussion

Understanding the infection process and disease cycle of a pathogen is fundamental for developing effective disease management strategies enabling targeted interventions during critical infection windows, thereby enhancing control efficacy [42]. The infection biology of *S. tanaceti* on pyrethrum is well characterised; the pathogen can originate from seedborne inoculum, causing symptoms in emerging seedlings or persisting as a latent infection within the host [12,13]. However, the disease cycle and infection process of *D. tanaceti* on pyrethrum remain largely unknown. In particular, the mode of entry, the timing of colonisation, and the host tissues colonised during early stages of disease development are not well characterised. The development of fluorescently labelled strains provides a valuable approach to investigate pathogen entry, colonisation, and early infection dynamics within host tissues.

Fluorescently labelled strains of *D. tanaceti* and *S. tanaceti* developed here provide a valuable tool for visualising early infection processes and tracking pathogen colonisation within host tissues. Most ATMT studies in filamentous fungi have focused on investigating gene function [18,19,43]. However, ATMT has also been used, albeit less frequently, to introduce fluorescent markers for studying infection dynamics and enabling visual differentiation of pathogens within plant tissues [14,16,22,44]. Plasmids pMAI31 and pMAI32 have previously been used to successfully transform *Leptosphaeria maculans* [45], demonstrating their utility for generating transgenic, fluorescently labelled fungal strains. In the context of *D. tanaceti*, where infection biology remains poorly characterised, this approach provides a means to directly observe early colonisation events that would otherwise be difficult to detect.

The recovery of predominantly single-copy T-DNA insertions from the assembled genome of transformed strains is consistent with previous reports of *Agrobacterium*-mediated transformation in filamentous fungi. Single-copy T-DNA insertions are generally preferred, as they are associated with more stable transgene expression and a reduced likelihood of gene silencing in filamentous fungi [46,47]. Although ATMT largely facilitates the random integration of T-DNA into the host genome and is often effective at producing single-copy insertions, multiple copies and tandem repeats can also occur [19,46]. For example, tandem insertions have been reported in *Didymella rabiei* [16], while both single and multiple insertions have been documented in *Didymella lentis* [22].

Partial T-DNA insertions and truncated integration events can occur during ATMT. The retention of essential elements required for fluorescent marker expression suggests that these constructs were likely functionally intact despite truncations [47–49]. The assembled genome sizes were slightly smaller than the estimated genome size. This discrepancy is commonly observed in short-read genome assemblies and typically attributed to the collapse or incomplete resolution of repetitive regions and low-complexity sequences like simple repeats [50,51]. Overall, the integration patterns fell within the range of outcomes reported for *Agrobacterium*-mediated transformation in closely related species.

For strain UOM DT1, T-DNA integration occurred either adjacent to or within predicted gene models, including loci associated with DNA repair functions. In one transformant, the insertion was located downstream of a Rad5-family gene (*rad5C*), a putative Rad5-like protein involved in error-free post-replication repair pathways, acting downstream of Rad18 to mediate template switching and replication fork restart [52–54]. Structural annotation suggests that the downstream insertion is unlikely to disrupt the open reading frame, which may explain the lack of observable effects on growth or morphology under the conditions tested. However, further analyses, such as RNA-seq or targeted genotoxic stress assays, would be required to determine whether gene expression was affected.

In contrast, in strain UOM DT5, T-DNA integration occurred within a gene predicted to encode a GMC oxidoreductase, suggesting disruption of the coding sequence in another transformant. The GMC oxidoreductase family comprises a diverse group of enzymes, including both secreted and intracellular members with metabolic functions [55,56], some of which have been associated with lignocellulose degradation and host colonisation in plant-pathogenic fungi [56,57]. Potentially, the absence of any detectable effect on growth rate or virulence following disruption of this locus could reflect functional redundancy with other GMC-family homologues in the genome or indicate that the disrupted gene encodes an intracellular enzyme not directly involved in pathogenicity.

Structural rearrangements, including inversions and deletions, were observed at T-DNA integration sites, highlighting the complexity of insertion events associated with *Agrobacterium*-mediated transformation. In particular, disruption of the GMC oxidoreductase (GMCox) open reading frame represents a more complex integration event compared with insertions occurring outside coding regions. Small deletions at T-DNA integration sites have been previously reported in both plants and filamentous fungi. While large-scale inversions and deletions at T-DNA integration sites are frequently documented [48,58], small deletions and inversions also occur [59,60], and may be more common than currently appreciated [61].

T-DNA insertions were also associated with genomic alterations in *S. tanaceti*, including deletions proximal to genes involved in key regulatory pathways. For instance, the deletion near a Rheb GTPase locus suggests potential effects on the TORC1 (Target of Rapamycin) signalling pathway, which plays a central role in regulating cell growth, nutrient sensing, and development in filamentous fungi [62,63]. Alterations in this region may influence transcriptional regulation, particularly if promoter or start codon regions are affected. In contrast, other insertion events occurred within regions annotated as hypothetical proteins without detectable structural rearrangements, illustrating the variability of integration outcomes and highlighting that ATMT can target diverse genomic regions and generate both simple and complex integration events.

Variation in fluorescence intensity among transformants likely reflects differences in T-DNA integration sites and species-specific factors influencing promoter activity and protein stability. Similar variability in fluorescence intensity of genome-integrated reporters has been reported in *Cochliobolus heterostrophus* [64] and *Nigrospora* sp. [36]. In contrast, fluorescence appeared more consistent among *S. tanaceti* transformants, suggesting more uniform expression of the reporter construct in this species. Despite these differences, the stable detection of fluorescence across all transformants indicates that the fluorescent proteins were functionally expressed, supporting the suitability of these strains for visualising infection processes.

The *in vitro* morphology and growth rates of most transformants were comparable to those of the wild type, suggesting that T-DNA insertion did not substantially affect genes required for these traits in the majority of cases. Variation in growth among transformants may be associated with the location of T-DNA integration within the genome [65,66]. Slower-growing strains were deemed unsuitable for further experiments because they would not accurately reflect the timing of the infection process and host-pathogen interaction *in planta*.

Although statistically significant differences in spore dimensions were detected among *D. tanaceti* isolates, mean conidial measurements remained broadly consistent with those previously reported for the species [67]. Similarly, conidial dimensions of *S. tanaceti* were generally consistent with previous descriptions, although conidia tended to be slightly smaller and narrower than those reported in the original species description [9]. Overall, these results indicate that genetic transformation had limited effects on conidial morphology for both species.

Direct epidermal penetration appears to be a predominant infection strategy of *D. tanaceti* during colonisation of pyrethrum leaves under the experimental conditions used. Similar infection patterns and timing between transformants and wild type strains in both *D. tanaceti* and *S. tanaceti* suggest that the transformation process did not substantially alter key aspects of pathogenic development. This is consistent with previous descriptions of the infection timeline of *S. tanaceti* in pyrethrum, where germination occurs at approximately 12 HAI and mesophyll invasion by around 54 HAI [41]

Direct epidermal penetration has also been reported in *S. tanaceti* [41], and in several necrotrophic and hemi-biotrophic fungi, including *Botrytis cinerea* and *Colletotrichum* spp., where penetration occurs either via appressoria or directly from hyphae without stomatal involvement [68,69]. Although stomatal penetration was not observed it cannot be excluded, particularly given that closely related species such as *Didymella rabiei* are capable of both direct epidermal and stomatal entry [16].

Glandular trichomes on the surface of pyrethrum leaves are known to exhibit intrinsic fluorescence across multiple wavelengths [70–73]. In addition, pathogen infection can induce the accumulation of fluorescent compounds and alter chlorophyll fluorescence, reflecting physiological changes associated with plant defence responses [74–76].

The overlap between plant autofluorescence and the emission spectra of fluorescent reporter proteins represents a common challenge in plant-pathogen imaging. In this context, the emission intensity of mNeonGreen and tdTomato appears sufficient to enable visual discrimination from the broader and weaker autofluorescence signals of plant tissues. This contrast is likely due to the narrow emission peaks and higher intensity of the reporter proteins compared with endogenous plant fluorescence.

Fluorescent *D. tanaceti* strains provide a valuable tool for investigating pathogen entry points and colonisation patterns across different plant tissues of pyrethrum. This approach may also help determine whether the pathogen exhibits systemic movement, as demonstrated for *Colletotrichum graminicola*, which spreads from roots to aerial tissues [17]. Similarly, GFP labelling of *A. lentis* enabled detailed characterisation of ascochyta blight progression in lentil [16]. Together, these studies illustrate the value of fluorescent protein reporters for elucidating infection biology in poorly characterised pathogens. Moreover, the use of distinct reporter genes such as *tdTomato* and *mNeonGreen* enables visual discrimination of each pathogen, providing a useful framework for future studies investigating co-infection dynamics.

Further work using whole-plant inoculation assays, quantitative disease assessments, and time-course analyses will be required to validate these observations under physiological conditions. In addition, quantitative approaches such as qPCR would provide valuable insight into pathogen biomass and colonisation dynamics, complementing qualitative observations. Such studies would contribute to a more comprehensive understanding of infection biology in pyrethrum, particularly given the limited information currently available for *D. tanaceti*.

## Supporting information

S1 Fig Schematic representation of plasmid pMAI31 carrying the mNeonGreen fluorescent protein gene used to transform *Didymella tanaceti.* (TIFF)

S2 Fig Schematic representation of plasmid pMAI32 carrying the tdTomato fluorescent protein gene used to transform *Stagonosporopsis tanaceti.* (TIFF)

S3 Fig Morphological characterisation of wild type and selected transformed strains of *Didymella tanaceti* after six days on potato dextrose agar. (A) Wild type strain BRIP 61988; (B-F) Transformed strains UOM DT1, UOM DT2, UOM DT3, UOM DT4 and UOM DT5.
(TIFF)

S4 Fig Morphological characterisation of wild type and selected transformed strains of *Stagonosporopsis tanaceti* after six days on potato dextrose agar. (A) Wild type strain UOM ST2; (B-E) Transformed strains UOM STC1R2, UOM STC2R2, UOM STC3R2 and UOM STC4R2.
(TIFF)

S1 Table Growth rates (mean ± SD, mm) of *Stagonosporopsis tanaceti* wild type (UOM ST2) and transformed strains (UOM STC1R2–UOM STC4R2) measured at Day 3, 6, and 9. Letters indicate statistically significant differences between strains within each time point based estimated marginal means (emmeans) with Tukey adjustment.
(DOCX)

S2 Table Growth rates (mean ± SD, mm) of *Didymella tanaceti* wild type (BRIP 61988) and transformed strains (UOM DT1–DT5) measured at Day 3, 6, 9, and 12. Letters indicate statistically significant differences between strains within each time point based on estimated marginal means (emmeans) with Tukey adjustment.
(DOCX)

S1 Protocol Potato dextrose agar (PDA), water agar (WA) and V8 media preparation. (DOCX)

S2 Protocol *Agrobacterium* mediated transformation induction media, antibiotics and overlay media preparation. (DOCX)
