# Supplementary material for: Agrobacterium tumefaciens-mediated transformation and T-DNA insertion characterisation of Didymella tanaceti and Stagonosporopsis tanaceti for infection visualisation in pyrethrum
Source: PLoS One. 2026 Aug 10;21(8):e0355715. doi: 10.1371/journal.pone.0355715 (PMC13456285; doi:10.1371/journal.pone.0355715)
Supplement: Supplementary file 2 [file pone.0355715.s011.pdf]

Dear Authors,

This manuscript presents a technically valuable output by developing fluorescently labelled strains of Didymella tanaceti and Stagonosporopsis tanaceti. The successful transformation and characterisation of these strains represent a methodological advance that will benefit the pyrethrum community. However, the manuscript has some major issues regarding the experimental design, data interpretation, and scientific writing that require revision before it meets the requirements for publication.

Major comments:

1. The manuscript contains substantial technical details. For example, the introduction section uses a full paragraph to introduce the development of fluorescence proteins, which are merely relevant to the main contribution of this manuscript. The discussion also follows the same pattern, focusing too much on technical details rather than the major findings.

2. The discussion includes detailed speculation about gene functions based on the homology searches and references from other organisms. Since whole-genome sequencing provides very useful information, the functions of these speculated genes still require additional evidence to support the findings. Therefore, the discussion of these genes in this context may be overstated and lacks sufficient experimental support.

3. In order to reflect the field condition, the authors applied a co-infection study that demonstrates proof-of-concept, but more measurements and background information should be addressed. The reasons for doing a co-infection study should provide more context. Also, in the manuscript, the "dynamics" of pathogen infection were mentioned but not demonstrated in the results. It would be better if the different time points post-inoculation in the results could align with the dynamics.

4. The formatting structure of the manuscript should be reconstructed.

Here are some technical issues noted:

a. Figure legends appear within the main text rather than at the end (Should be place at the end after references)

b. Some formatting inconsistencies with uints and spacing

For example, line 50 is "60 ℃", but line 84 "21℃" does not have a space.

c. Orphaned annotation symbols apear in the text (lines 417-419)

5. Since most of the results are observations instead of quantification, it is recommended that the numbers of observations and experiment repeat numbers should be addressed.

Minor comments

1. Consider briefly noting why fluorescent markers are particularly valuable for studying **co-infections.**

2. Ensure all species names are properly italicised in both the main text and figure legends.

3. Can any other fluorescence staining be applied to observe the wild-type strains *in situ*?

This work makes a solid contribution to the methodological toolkit available for pyrethrum pathology research. The technical quality is high, and the potential applications are clear. With attention to the presentation and organisation suggestions above, this manuscript will serve as a valuable resource for researchers working with these important plant pathogens.

The development of these fluorescent strains represents a meaningful step forward in our ability to study these pathogens for which research tools have been limited.
