# Supplementary material for: Agrobacterium tumefaciens-mediated transformation and T-DNA insertion characterisation of Didymella tanaceti and Stagonosporopsis tanaceti for infection visualisation in pyrethrum
Source: PLoS One. 2026 Aug 10;21(8):e0355715. doi: 10.1371/journal.pone.0355715 (PMC13456285; doi:10.1371/journal.pone.0355715)
Supplement: Supplementary file 3 [file pone.0355715.s005.docx]

**S1 Table**. Growth rates (mean ± SD, mm) of *Stagonosporopsis tanaceti* wild type (UOM ST2) and transformed strains (UOM STC1R-UOM STC4R2) measured at Day 3, 6, and 9. Letters indicate statistically significant differences between strains within each time point based estimated marginal means (emmeans) with Tukey adjustment.

| **Day** | **Strain** | **mean_sd** |
| --- | --- | --- |
| Day3 | UOM ST2 | 11.7 ± 0.6   b |
|  | UOM STC1R2 | 10.7 ± 0.6  ab |
|  | UOM STC2R2 | 10.3 ± 1.2  a |
|  | UOM STC3R2 | 10.7 ± 0.6  ab |
|  | UOM STC4R2 | 10.7 ± 0.6  ab |
| Day6 | UOM ST2 | 24.7 ± 0.6  a |
|  | UOM STC1R2 | 23.7 ± 0.6  a |
|  | UOM STC2R2 | 24.7 ± 0.6  a |
|  | UOM STC3R2 | 24.7 ± 0.6  a |
|  | UOM STC4R2 | 24 ± 0  a |
| Day9 | UOM ST2 | 35 ± 0  a |
|  | UOM STC1R2 | 35 ± 0  a |
|  | UOM STC2R2 | 35.3 ± 0.6  a |
|  | UOM STC3R2 | 35 ± 0  a |
|  | UOM STC4R2 | 34.7 ± 0.6  a |
