# Supplementary material for: Agrobacterium tumefaciens-mediated transformation and T-DNA insertion characterisation of Didymella tanaceti and Stagonosporopsis tanaceti for infection visualisation in pyrethrum
Source: PLoS One. 2026 Aug 10;21(8):e0355715. doi: 10.1371/journal.pone.0355715 (PMC13456285; doi:10.1371/journal.pone.0355715)
Supplement: Supplementary file 4 [file pone.0355715.s006.docx]

**S2 Table.** Growth rates (mean ± SD, mm) of *Didymella tanaceti* wild type (BRIP 61988) and transformed strains (UOM DT1-DT5) measured at Day 3, 6, 9, and 12. Letters indicate statistically significant differences between strains within each time point based on estimated marginal means (emmeans) with Tukey adjustment.

| **Day** | **Strain** | **mean_sd** |
| --- | --- | --- |
| Day3 | BRIP 61988 | 7 ± 0   b |
|  | UOM DT1 | 6.3 ± 0.6   b |
|  | UOM DT2 | 6 ± 1   b |
|  | UOM DT3 | 6.3 ± 0.6   b |
|  | UOM DT4 | 3 ± 0  a |
|  | UOM DT5 | 7 ± 0   b |
| Day6 | BRIP 61988 | 15.7 ± 0.6   b |
|  | UOM DT1 | 15.7 ± 0.6   b |
|  | UOM DT2 | 15.7 ± 1.2   b |
|  | UOM DT3 | 15.3 ± 0.6   b |
|  | UOM DT4 | 10 ± 0  a |
|  | UOM DT5 | 15.3 ± 0.6   b |
| Day9 | BRIP 61988 | 22.7 ± 0.6   b |
|  | UOM DT1 | 23.7 ± 0.6   b |
|  | UOM DT2 | 23.7 ± 0.6   b |
|  | UOM DT3 | 23.3 ± 1.2   b |
|  | UOM DT4 | 16 ± 0  a |
|  | UOM DT5 | 23.3 ± 0.6   b |
| Day12 | BRIP 61988 | 30.3 ± 1.5   b |
|  | UOM DT1 | 32 ± 0   b |
|  | UOM DT2 | 31.7 ± 0.6   b |
|  | UOM DT3 | 30.7 ± 2.3   b |
|  | UOM DT4 | 23 ± 0  a |
|  | UOM DT5 | 31.3 ± 0.6   b |
