# Supplementary material for: Agrobacterium tumefaciens-mediated transformation and T-DNA insertion characterisation of Didymella tanaceti and Stagonosporopsis tanaceti for infection visualisation in pyrethrum
Source: PLoS One. 2026 Aug 10;21(8):e0355715. doi: 10.1371/journal.pone.0355715 (PMC13456285; doi:10.1371/journal.pone.0355715)
Supplement: Supplementary file 5 [file pone.0355715.s007.docx]

**S1 Protocol. Potato dextrose agar (PDA), water agar (WA) and V8 media preparation**

**PDA** was prepared by weighing 37 g of commercial PDA powder (Difco™, BD, Sparks, MD, USA) and dissolving it in 1 L of RO water. The mixture was stirred until fully homogenised and then autoclaved at 121 °C for 20 minutes.

WA was prepared by adding 20 g of agar powder (WA; Ajax Finechem, Thermo Fisher Scientific, Australia) to 1 L of RO water. Upon mixing, the media was autoclaved at 121 °C for 20 minutes. Similarly, Potato Dextrose Agar (PDA) media was prepared by weighing 37 g of commercial PDA powder (DifcoTM) and adding it to 1 L of RO water. The mixture was shaken until uniformity and autoclaved for 20 minutes at 121 °C.

V8 juice agar (V8; Campbell Soup Company, Lemnos, VIC, Australia) was prepared by adding 200 ml of V8 vegetable juice filtered with a cheesecloth to 800 ml of RO water. Drops of 10 M NaOH were added to the mixture until a pH of 6.25 was achieved. Then, 20 g of agar were added, shaken until uniform and autoclaved at 121 °C for 20 minutes.
