# Supplementary material for: Agrobacterium tumefaciens-mediated transformation and T-DNA insertion characterisation of Didymella tanaceti and Stagonosporopsis tanaceti for infection visualisation in pyrethrum
Source: PLoS One. 2026 Aug 10;21(8):e0355715. doi: 10.1371/journal.pone.0355715 (PMC13456285; doi:10.1371/journal.pone.0355715)
Supplement: Supplementary file 6 [file pone.0355715.s008.docx]

##### **S2 Protocol. *Agrobacterium* mediated transformation induction media, antibiotics and overlay media preparation**

##### Cefotaxime, hygromycin and acetosyringone preparation.

A 100 mg/mL cefotaxime stock solution was prepared, filter-sterilized, and stored at 4 °C. Hygromycin was used as a pre-made 50 mg/mL stock solution throughout the experiment. Acetosyringone was prepared as a 10 mM stock by dissolving 0.0196 g in 500 µL of dimethyl sulfoxide (DMSO) (Sigma Aldrich, USA). Short-term storage was at 4 °C, and long-term storage was at −21 °C.

Table 1. MM salts for induction media (IM)

| **Formula** | **Chemical Name** | **Amount per litre (g)** |
| --- | --- | --- |
| KH₂PO₄ | Potassium dihydrogen phosphate | 3.625 |
| K₂HPO₄ | Dipotassium hydrogen phosphate | 5.125 |
| NaCl | Sodium chloride | 0.375 |
| MgSO₄·7H₂O | Magnesium sulfate heptahydrate | 1.250 |
| CaCl₂·2H₂O | Calcium chloride dihydrate | 0.165 |
| FeSO₄·7H₂O | Ferrous sulfate heptahydrate | 0.0062 |
| (NH₄)₂SO₄ | Ammonium sulfate | 1.250 |

*Note: each salt was entirely dissolved before adding the next one*

##### Preparation of 1M MES for IM

1 M MES buffer (pH 5.3) was prepared to use in Induction Media (IM) by dissolving 7.7 g of MES (2-(N-morpholino) ethanesulfonic acid) in approximately 45 mL of distilled water. The pH was adjusted to 5.3 using 5 M KOH, and the solution was brought to a final volume of 50 mL with distilled water. The buffer was filter sterilised and added directly to molten IM once the medium had cooled to ~50 °C.

##### IM media preparation

To prepare 1 L of IM agar, 400 mL of 2.5× MM salts, 0.6 g of glucose, 5 mL of glycerol, 545 mL of distilled water, and 20 g of agar were mixed. The media was autoclaved at 121 °C for 20 minutes. Once cooled to 50 °C, 50 mL of sterile 1 M MES stock (pH 5.3) and 500 µL of acetosyringone (AS) were added. After mixing, the media was poured Petri plates and stored at 4 °C as required.

##### Overlay media preparation

The overlay medium for transformation, was V8 media for *S. tanaceti* and PDA for *D. tanaceti*. After autoclaving, the media was cooled to approximately 50 °C. Then, cefotaxime and hygromycin were added to achieve final concentrations of 100 µg/mL and 50 µg/mL, respectively. This was done by adding 200 µg/mL cefotaxime stock solution and 100 µg/mL hygromycin stock solution at a 1:1,000 dilution into the molten medium. The medium was mixed gently but thoroughly before being poured into Petri plates.
