# Supplementary material for: Agrobacterium tumefaciens-mediated transformation and T-DNA insertion characterisation of Didymella tanaceti and Stagonosporopsis tanaceti for infection visualisation in pyrethrum
Source: PLoS One. 2026 Aug 10;21(8):e0355715. doi: 10.1371/journal.pone.0355715 (PMC13456285; doi:10.1371/journal.pone.0355715)
Supplement: Supplementary file 11 [file pone.0355715.s016.docx]

Dear reviewers,

We thank the reviewers and editor for their thorough evaluation of our manuscript and for their constructive comments. These suggestions have further improved the clarity, structure, and focus of the study. All comments have been carefully considered and addressed, as detailed below:

| Comment | Response |
| --- | --- |
| 1. I suggest to do some minor corrections: Transformant UOMSTC1R2 is mentioned in two ways: UOM STC1R2 and UOMSTC1R2 | Thank you for your comment. The transformant name has now been corrected in the text, adding the space for consistency. |
| 2. Jellyfish v.2.3.1, and you have also mentioned the following: The estimated genome size for D. tanaceti isolates was 40.7 Mbp using Jellyfish. Here 'Jellyfish' means the software, doesn't it? So, please mention the software with an appropriate version throughout the manuscript | Thank you very much for the comment. The version of Jellyfish (software) has been added throughout the text. |
| 3. in the introduction you have mentioned the following: The green fluorescent protein (GFP), derived from the Aequorea victoria jellyfish, is a highly stable reporter capable of resisting temperatures over 60°C, low pH and proteases. This statement indicates that GFP is derived from jellyfish. | Thank you for the comment. The wording in the introduction has been changed accordingly to avoid confusion. |

We believe that these revisions have significantly improved the clarity, focus, and scientific rigour of the manuscript. We thank the reviewers again for dedicating their valuable time and detailed feedback, and hope that the revised version meets the expectations for publication.

Kind regards,

Violeta Carrillo
