# Supplementary material for: Agrobacterium tumefaciens-mediated transformation and T-DNA insertion characterisation of Didymella tanaceti and Stagonosporopsis tanaceti for infection visualisation in pyrethrum
Source: PLoS One. 2026 Aug 10;21(8):e0355715. doi: 10.1371/journal.pone.0355715 (PMC13456285; doi:10.1371/journal.pone.0355715)
Supplement: Supplementary file 12 [file pone.0355715.s009.docx]

**Title of the Study:** Elucidating pathogen interactions in *Tanacetum cinerariifolium* (pyrethrum) using fluorescently labelled *Didymella tanaceti* and *Stagonosporopsis tanaceti*.

**Overall Comments:**

The article is thorough, well-organized, and clearly presents data on transformation efficiency, fluorescence validation, morphological assessment, molecular characterization, and pathogenicity comparisons. The incorporation of microscopy, quantitative fluorescence analysis (CTCF), genome assembly statistics, BUSCO completeness assessments, and T-DNA insertion mapping effectively supports the study's findings. Nonetheless, certain areas would benefit from clarification, consistency corrections, grammatical improvements, and enhanced data presentation to further strengthen scientific clarity and readability.

- Confidence intervals or error bars (SD/SE) should be specified in figure legends.
- The p-value range reported for *D. tanaceti* (p = 0.02 – 0.0002) is unclear. Exact p-values should be reported for each comparison.
- You mentioned that 15 ROIs per transformant, need to include the number of biological replicates
- Some paragraphs are overly detailed, particularly: T-DNA rearrangements discussion, autofluorescence explanation and literature comparisons.
- Improve explanation of findings and discussion
- Include proper units and legends
- Condense repetitive statements and tighten literature comparison sections.
- Briefly reinforce statistical validation if performed.
