# Supplementary material for: Agrobacterium tumefaciens-mediated transformation and T-DNA insertion characterisation of Didymella tanaceti and Stagonosporopsis tanaceti for infection visualisation in pyrethrum
Source: PLoS One. 2026 Aug 10;21(8):e0355715. doi: 10.1371/journal.pone.0355715 (PMC13456285; doi:10.1371/journal.pone.0355715)
Supplement: Supplementary file 14 [file pone.0355715.s014.docx]

Dear reviewers,

We thank the reviewers and editor for their thorough evaluation of our manuscript and for their constructive comments. These suggestions have significantly improved the clarity, structure, and focus of the study. All comments have been carefully considered and addressed, as detailed below:

| **Comment** | **Response** |
| --- | --- |
| *“Are these fluorescently labelled strains of both D. tanaceti and S. tanaceti natural isolates or registered fungal accessions?”* | We thank the reviewer for this question. Stagonosporopsis tanaceti isolate UOM ST2 is a natural isolate, whereas Didymella tanaceti strain BRIP 61988 is a registered accession. The corresponding reference describing BRIP 61988 has been included at its first mention in the manuscript (Page 4, line 126). |
| “*Which type of a* Agrobacterium tumefaciens *strain was used for transformation? in some places the genus species Stagonosporopsis tanaceti was not italicized? Please italicize it*” | The Agrobacterium tumefaciens strain used for transformation has now been specified in the main text (Page 6, line 180). In addition, all occurrences of Stagonosporopsis tanaceti have been carefully revised and are now consistently italicised throughout the manuscript. |
| *“Are there any naturally resistant lines of Tanacetum cinerariifolium, or does any study show the natural resistance found against any of these fungal pathogens?”* | To our knowledge, there are currently no well-documented naturally resistant lines of Tanacetum cinerariifolium against these pathogens. Pyrethrum breeding programmes have historically focused on traits such as pyrethrin yield rather than disease resistance. |
| “*The manuscript overemphasises pathogen interactions. The title, abstract, and discussion should better reflect the main contribution*” | We thank the reviewer for this comment. The manuscript has been substantially revised to better reflect its primary focus. The title, abstract, and discussion have been reframed to emphasise the development and characterisation of fluorescent transformants and their application for visualisation in planta, rather than suggesting a comprehensive analysis of pathogen interactions. Statements implying detailed interaction analysis have been removed or moderated, and the infection observations are now presented as an initial descriptive framework. |
| *“After sequencing the strains and confirming the T DNA using BLASTN, have you tried to find out the number of integrated copies of the T DNA? If so, how many copies of the T-DNA were found?”* | We thank the reviewer for this comment. The number of T-DNA copies was assessed using BLAST-based analysis against the assembled genomes, as described in the Methods. Based on this approach, all transformants analysed contained a single complete T-DNA insertion. This has now been clarified in the Results section (Page 17, Lines 598-602) |
| ***“****What kinds of genes were predicted using AUGUSTUS with the Botrytis cinerea reference? Have you predicted the fluorescent-related proteins alone or any more candidate genes and their proteins? If so, could you please list them as a table with candidate gene IDs****”*** | We thank the reviewer for this comment. Gene prediction using AUGUSTUS was performed to identify gene models in regions surrounding T-DNA insertion sites. As AUGUSTUS output in Geneious did not provide functional annotation, predicted genes were further analysed using BLAST to infer putative functions. Analyses were restricted to genes associated with insertion sites rather than genome-wide predictions. A supplementary table has been added summarising these genes, including AUGUSTUS gene IDs and BLAST-based annotations. |
| *“Pathogen biomass and colonization dynamics should be validated using more precise techniques such as quantitative PCR (qPCR) or RT-qPCR. This is crucial to provide a definitive quantification of fungal load that complements the qualitative fluorescence data”* | We thank the reviewer for this suggestion. We agree that quantitative approaches such as qPCR or RT-qPCR would provide valuable insights into pathogen biomass and colonisation dynamics. However, these analyses were beyond the scope of the present study, which focuses on the generation and characterisation of fluorescent transformants and qualitative description of infection processes. A statement has been added to the Discussion highlighting this as an important direction for future work (Page 27, Line 1179) |
| **Co-infection and interaction claims** | We thank the reviewer for these comments. All sections related to pathogen-pathogen interactions and co-infection analysis have been removed from the manuscript to avoid overinterpretation. The study has been refocused to emphasise the development of fluorescent strains and their application for visualisation. Observations of co-localisation are now presented only as preliminary and descriptive, demonstrating feasibility rather than biological interaction. |
| *“Some findings were hidden in the discussion section: Lines 558-567 (epidermal penetration), 570-581 (infection intervals), 583-596 (co-infection observations), and 598-618 (autofluorescence characterisation) include primary data that should be in findings rather than discussion”* | We thank the reviewer for this important observation. Sections previously containing primary observations in the Discussion have been revised. Relevant findings (e.g., infection timing, co-localisation observations, and autofluorescence characterisation) have been moved or appropriately integrated into the Results section. The Discussion has been restructured to focus on biological interpretation rather than presentation of results. |
| *“The discussion section is long and should be reduced or shifted to additional material, particularly thorough technical validation details that do not increase biological knowledge”* | We thank the reviewer for this suggestion. The Discussion has been substantially shortened and restructured to remove excessive technical detail and improve focus on biological interpretation. |
| *“The manuscript has certain key methodological deficiencies: In terms of statistical analysis and experimental design, Line 155 lacks software specifications, alpha values, and ANOVA assumption testing. Because of the small sample numbers used in detached leaf tests, power analysis must be justified”* | We thank the reviewer for this comment. Statistical methods have been clarified, including software specifications, significance threshold (α = 0.05), and assumption testing. The detached leaf assay has been explicitly described as a qualitative, exploratory experiment rather than a fully powered quantitative analysis, and now this has been clarified in the Methods. |
| ***“****The paper does not give any information on the replication structure (Biological and technical replicates)”* | We thank the reviewer for this comment. The replication structure has now been clarified in the Methods section. Each leaflet was treated as an independent biological replicate, and fluorescence measurements consisted of technical replication (multiple ROIs) within a single biological sample. |
| *“Furthermore, the detached leaf test technique is insufficient to validate biological equivalency due to the absence of quantitative disease severity score and no entire plant validation beyond detached leaves especially in the context of pathogens interaction in-planta”* | We thank the reviewer for this comment. The manuscript has been revised to clarify that the detached leaf assay was used as a controlled, qualitative system to describe early infection processes rather than to assess quantitative disease severity or full biological equivalence. The limitations of this approach and the need for future whole-plant studies have been explicitly acknowledged in the Discussion. |
| *“There are various limits to genome analysis. The assembly quality measures are lacking, as they lack N50 values and contig counts. If feasible, I would consider comparing the BUSCO data to other Didymellaceae genomes. Genome statistics (lines 322-329) should be tabulated rather than included in the text”* | We thank the reviewer for this suggestion. Genome statistics, including assembly size, contig counts, and N50 values, have now been tabulated for clarity (Page 16). BUSCO results have been retained in the text. This improves presentation and facilitates comparison across strains. |
| *“Given the experimental methodology, the result on direct epidermal penetration (line 558) seems dubious. Within hours, detached leaves lose their stomatal function, which may make epidermal penetration the only viable route. To confirm biological results about natural infection methods, this methodological bias has to be validated using intact plants and functioning stomata”* | We thank the reviewer for this insightful comment. We acknowledge that detached leaf assays may influence stomatal function. The Discussion has been revised to clarify that observations of direct epidermal penetration are limited to the experimental conditions used (Page 26, Line 1061) and do not exclude alternative infection routes. |
| *“This study would be greatly boosted if it were repositioned as the basis for future biological research. Expand the biological characterization with these verified techniques and transfer comprehensive transformation methods to supplemental material. Although the fluorescent strains are useful research tools, they require significant refinement in their biological application before they can be published”* | We thank the reviewer for this perspective. The manuscript has been refocused to emphasise the development and validation of fluorescent transformants, while presenting infection observations as an initial descriptive framework to support future biological studies. |
| ***Figure captions: request to remove from main text*** | Figure captions have been retained in the main manuscript text in accordance with PLOS ONE guidelines, which state that “figure captions should be placed in the manuscript text in read order, immediately following the paragraph where the figure is first cited.” Supplementary figures and tables have been placed after the references, as recommended. |
| ***Supplementary materials and media composition:*** | We thank the reviewer for this comment. The full composition of all media has been provided in the Supplementary Materials, including ingredient concentrations and preparation steps. Manufacturer details have been included at first mention in the manuscript. |
| *“Why is D. tanaceti the more important?”* | We thank the reviewer for this comment. Context of the disease now added as part of the introduction (lines 60-70) |
| *This reads more like a review on fluorescent proteins than necessary background for this study. You can compress it a little bit."* | We thank the reviewer for this comment. Introductory section on fluorescent proteins has now been compressed (lines 73-114) |
| *“what type of light? shelf, BOD"* | We thank the reviewer for this comment. The type of light source has now been clarified by specifying the growth chamber used for incubation in the Methods section (lines 144-146) |
| *"Clarify whether kanR remains in the final constructs or was replaced".* | We thank the reviewer for this comment. Now clarified (line 153) |
| *Write in standard fromat (Optical density 600)* | We thank the reviewer for this comment, standard format now added (line 181) |
| *“Briefly justify why different purification methods were used to”* | We thank the reviewer for this comment, detail now added in lines 214-215 |
| *clarify ROI unit.* | We thank the reviewer for this comment. ROI unit is now clarified (line 240-241) |
| *Size of agar plug subcultures* | We thank the reviewer for this comment. Agar plug size now clarified (Line 258) |
| *“What was the DNA template concentration”* | We thank the reviewer for this comment. DNA concentrations were measured using a Qubit fluorometer. As no PCR or reaction-based amplification steps were performed prior to sequencing, no template DNA concentration per reaction was applicable. |
| *Replace integration for RETENTION. Up to now, fluorescence retention demonstrates stable expression, but it has not necessarily proven genomic integration.* | We thank the reviewer for this comment. Replaced with correct wording (line 451) |
| *“Please either provide exact p-values per comparison or report overall ANOVA p-value and Tukey grouping"* | We thank the reviewer for this comment. Exact p values now provided as part of the results (Lines 488-493) |
| *How many biological replicates were used to assess colony growth rate?*  *Was radial growth measured quantitatively or visually?* | We thank the reviewer for this comment. Number of replicates is specified in methods (line 257). Radial growth was measured quantitatively data provided in supplementary table |
| *Why are transformed assemblies slightly smaller than the estimated genome size?* | We thank the reviewer for this comment. This has now been added as part of the discussion (Lines 895-898) |
| *Were lesion sizes measured quantitatively?*  *Was disease severity statistically compared?* | We thank the reviewer for this comment. Lesion size and disease severity were not quantified in this study, as the primary objective was to assess the feasibility of using fluorescently labelled strains to visualise infection and colonisation processes. The observations presented are therefore qualitative and intended to provide an initial description of infection dynamics. Quantitative assessment of disease severity and lesion development represents an important direction for future work. |
| *Was this based on replicated observations? If yes, specify the number of replicates* | We thank the reviewer for this comment. Number of replicates now added to manuscript (line 695) |
| *Was any autofluorescence control performed (mock-inoculated leaves under DsRed filter)?*  *Were exposure settings standardised between wild type and transformant?*  *I am asking about false positives due to autofluorescence* | We thank the reviewer for this comment. Yes, autofluorescence controls were considered during image acquisition. Mock-inoculated leaf tissues were examined under the DsRed and YFP filter sets to assess background fluorescence. Exposure settings were standardised across all images (1 s exposure) to ensure comparability between wild-type and transformant samples.  To minimise false positives, fluorescence signals were cross-checked between channels. Signals observed under the DsRed filter were also examined under the YFP filter; persistent fluorescence across both channels was interpreted as plant autofluorescence, whereas signals restricted to a single channel were attributed to the fluorescent protein. The same approach was applied for Didymella tanaceti transformants.  While representative images of these controls were not retained, this comparative approach was consistently used to distinguish true fluorescence from background autofluorescence |
| *In the discussion section: Can you state explicitly what was previously unknown about D. tanaceti (entry mode? colonisation timing? tissue specificity?) to sharpen the gap further?* | We thank the reviewer for this comment. Specific gap now added (line 829-831) |
| *Could copy number differences explain intensity variation?* | We thank the reviewer for this comment. Differences in fluorescence intensity are unlikely to be explained by variation in T-DNA copy number, as all selected transformants were confirmed to contain a single insertion. However, variation in fluorescence intensity may be influenced by the genomic location of the T-DNA insertion |
| *Do you have preliminary evidence of systemic movement? Or is this purely prospective?* | We thank the reviewer for this comment. At this stage, there is no direct evidence supporting systemic movement of the pathogen in this study. The suggestion is therefore prospective and based on observations reported in other plant–pathogen systems. |
| *Either use DAI or HAI* | We appreciate the reviewer’s comment regarding unit standardisation. In this study, hours after inoculation (HAI) were used to describe early infection events observed microscopically (e.g. spore germination, germ tube emergence, and penetration), which occur over short timeframes. In contrast, days after inoculation (DAI) were used to describe later-stage processes such as symptom development and tissue colonisation, which occur over longer periods. This distinction was applied consistently throughout the manuscript to reflect the biological scale of the observations. |

We believe that these revisions have significantly improved the clarity, focus, and scientific rigour of the manuscript. We thank the reviewers again for dedicating their valuable time and feedback, and hope that the revised version meets the expectations for publication.
