# Supplementary material for: Agrobacterium tumefaciens-mediated transformation and T-DNA insertion characterisation of Didymella tanaceti and Stagonosporopsis tanaceti for infection visualisation in pyrethrum
Source: PLoS One. 2026 Aug 10;21(8):e0355715. doi: 10.1371/journal.pone.0355715 (PMC13456285; doi:10.1371/journal.pone.0355715)
Supplement: Supplementary file 15 [file pone.0355715.s015.docx]

Dear reviewers,

We thank the reviewers and editor for their thorough evaluation of our manuscript and for their constructive comments. These suggestions have further improved the clarity, structure, and focus of the study. All comments have been carefully considered and addressed, as detailed below:

| Comment | Response |
| --- | --- |
| 1. Line 18 insert a period after losses and start a new sentence with However. | Thank you for the comment on sentence structure. The error has been amended |
| 2. Line 60 Insert a period after tissues and start a new sentence with However. | Thank you for the comment. The error was in line 61, and is now amended |
| 3. Line 100 what was the pore size of the millipore? Express HAI first as Hours after infection. Why were the cultures sometimes incubated under light and sometimes under light/darkness. | The pore size of the Miracloth used for filtration has now been added to the Materials and Methods section (22-25 µm).  Regarding the use of the term "inoculation", we have retained this terminology throughout the manuscript. Inoculation refers to the introduction of the pathogen onto the host, whereas infection refers to the subsequent establishment and colonisation of host tissues. As infection is not necessarily confirmed at the time of pathogen application, we consider "inoculation" to be the most appropriate term in this context.  Thank you for picking this up. The incubation condition reported in line 144 has been corrected to darkness. This incubation step was conducted in a different laboratory associated with work involving genetically modified microorganisms at the university. Consequently, the incubation conditions at this stage differed from those used in subsequent experiments. |
| 4. Line 185 How many plates were measured to get the average colony diameter. | Thank you very much for your comment. Average colony diameter was measured from three replicate plates for each isolate. This information is provided in the Materials and Methods section (Line 188). |
| 5. Line 502 Appropriate reference should be cited here. You wondered if the pathogen could enter through the stomata did you try to infect the leaves on the lower epidermis where most stomata are located? | Thank you for your comment. We believe there may be a discrepancy between the comment and the cited line number. Line 502 (now line 544) refers to the discussion of T-DNA integration sites and the putative function of the Rad5-family DNA repair protein, which is supported by references 52-54. We did find an error in Line 566:  “Glandular trichomes on the surface of pyrethrum leaves are known to exhibit intrinsic fluorescence across multiple wavelengths [68-70]. [71]”).  This has now been corrected. We would be very grateful if the reviewer could clarify the intended section if a different passage was being referred to.  We have clarified in the Discussion (Line 612) that potential penetration through stomata was not directly observed in this study and remains speculative. This has been identified as an area requiring further investigation. |
| 6. The quality of figure 2 is quite poor. The resolution should be improved, also check the format required for formal publication. | Thank you for your comment. Figure 2, as well as other figures has been reformatted and resubmitted in TIFF format at 300 dpi. The figure dimensions have also been adjusted to comply with the image size and resolution requirements specified by PLOS ONE for publication. |
| 7. Line 163-166, add the fluorescence quantification method and the information of dichroic filters. The emission filters are long-pass or band-pass ones? | Thank you for your comment: Fluorescent filters information is now added into the paragraph for completeness. Emission filters are band-pass. Fluorescent quantification description and formula is located from line 169 to 185. |
| 8. Lines 336-339. Why was spore size measurement only for D. tanaceti but not for S. tanaceti? It should be determined if there is statistical difference in spore size between wild-type and transformed strains. Besides, the sporulation speed and amount between wild-type and transformed strains are better to be compared since they often affect fungal virulence. | Thank you for this suggestion. Additional conidial measurements were conducted for both *D. tanaceti* and *S. tanaceti* isolates. Conidial length and width were measured for the wild-type and transformed strains, and statistical comparisons were performed using one-way ANOVA followed by pairwise comparisons of estimated marginal means. The Methods, Results, and Discussion sections have been updated accordingly. For *S. tanaceti*, conidial measurements were obtained from the wild type isolate UOM ST2 and the transformed isolate UOMSTC1R2, as these were the isolates sporulating at the time of assessment  Comparison of sporulation speed and sporulation quantity was not undertaken in the present study. Sporulation in *S. tanaceti* is normally difficult and inconsistent in non-transformed isolates. In this study, sporulation was very variable and inconsistent among both wild type and transformed isolates under the conditions tested, making reliable quantitative comparisons difficult. |
| 9. Since the transformed strains are intended to be used in pathological study, the comparable virulence of wild-type and transformed strained strains on hosts is required. The analysis in fungal biomass and plant lesion developments would help. | Thank you for this suggestion. We agree that quantitative assessments of virulence, including lesion development and fungal biomass measurements, would provide additional validation of the biological equivalence of transformed and parental strains. However, the primary objective of this study was the generation and characterisation of fluorescent transformants for future infection biology studies. During pathogenicity assays, transformed strains successfully infected host tissue and produced lesions comparable to those observed for the parental strains, with no obvious differences in symptom development. Furthermore, the manuscript already acknowledges the need for future whole-plant inoculation assays, quantitative disease assessments and qPCR-based biomass quantification to further investigate pathogen colonisation and disease development under physiological conditions. |
| 10. Lacking the medium formulas used in transformant construction. | Thank you for the feedback. Added formulas for Lysogeny Broth and Induction Media in the main text and further preparation is contained in Supplementary material |
| 11. Line 126, Liquid cultures of Agrobacterium strain EHA105 “in LB broth. Add LB medium formula. | Thank you for the feedback. Now added in text |
| 12. Line 129, Add the formula of induction medium. | Thank you for the feedback. Now added in text |

We believe that these revisions have significantly improved the clarity, focus, and scientific rigour of the manuscript. We thank the reviewers again for dedicating their valuable time and detailed feedback, and hope that the revised version meets the expectations for publication.

Kind regards,

Violeta Carrillo
