# Supplementary material for: Agrobacterium tumefaciens-mediated transformation and T-DNA insertion characterisation of Didymella tanaceti and Stagonosporopsis tanaceti for infection visualisation in pyrethrum
Source: PLoS One. 2026 Aug 10;21(8):e0355715. doi: 10.1371/journal.pone.0355715 (PMC13456285; doi:10.1371/journal.pone.0355715)
Supplement: Peer review history

# Peer review: Decision Letter 0

**DOI:** 10.1371/journal.pone.0355715.r001

**Authors:** Karthikeyan Thiyagarajan

24 Mar 2026

Dear Dr. Vaghefi,

Thank you for submitting your manuscript to PLOS ONE. After careful consideration, we feel that it has merit but does not fully meet PLOS ONE’s publication criteria as it currently stands. Therefore, we invite you to submit a revised version of the manuscript that addresses the points raised during the review process.

Please submit your revised manuscript by May 08 2026 11:59PM. If you will need more time than this to complete your revisions, please reply to this message or contact the journal office at plosone@plos.org. When you're ready to submit your revision, log on to https://www.editorialmanager.com/pone/ and select the 'Submissions Needing Revision' folder to locate your manuscript file.

- A letter that responds to each point raised by the academic editor and reviewer(s). You should upload this letter as a separate file labeled 'Response to Reviewers'.
- A marked-up copy of your manuscript that highlights changes made to the original version. You should upload this as a separate file labeled 'Revised Manuscript with Track Changes'.
- An unmarked version of your revised paper without tracked changes. You should upload this as a separate file labeled 'Manuscript'.

If you would like to make changes to your financial disclosure, please include your updated statement in your cover letter. Guidelines for resubmitting your figure files are available below the reviewer comments at the end of this letter.

If applicable, we recommend that you deposit your laboratory protocols in protocols.io to enhance the reproducibility of your results. Protocols.io assigns your protocol its own identifier (DOI) so that it can be cited independently in the future. For instructions see: https://journals.plos.org/plosone/s/submission-guidelines#loc-laboratory-protocols. Additionally, PLOS ONE offers an option for publishing peer-reviewed Lab Protocol articles, which describe protocols hosted on protocols.io. Read more information on sharing protocols at https://plos.org/protocols?utm_medium=editorial-email&utm_source=authorletters&utm_campaign=protocols.

As the corresponding author, your ORCID iD is verified in the submission system and will appear in the published article. PLOS supports the use of ORCID, and we encourage all coauthors to register for an ORCID iD and use it as well. Please encourage your coauthors to verify their ORCID iD within the submission system before final acceptance, as unverified ORCID iDs will not appear in the published article. *Only*  the individual author can complete the verification step; PLOS staff *cannot*  verify ORCID iDs on behalf of authors.

We look forward to receiving your revised manuscript.

Kind regards,

Karthikeyan Thiyagarajan, PhD

Academic Editor

PLOS One

Journal Requirements:

When submitting your revision, we need you to address these additional requirements.

1. Please ensure that your manuscript meets PLOS ONE's style requirements, including those for file naming. The PLOS ONE style templates can be found at

https://journals.plos.org/plosone/s/file?id=wjVg/PLOSOne_formatting_sample_main_body.pdf and

https://journals.plos.org/plosone/s/file?id=ba62/PLOSOne_formatting_sample_title_authors_affiliations.pdf

2. Thank you for stating the following in the Acknowledgments Section of your manuscript: [This research was supported by the University of Melbourne Scholarship and Botanical Resources Australia Pty Ltd. Thanks to Camilla Langlands-Perry for her support during the transformation processes.]

We note that you have provided funding information that is not currently declared in your Funding Statement. However, funding information should not appear in the Acknowledgments section or other areas of your manuscript. We will only publish funding information present in the Funding Statement section of the online submission form.

Please remove any funding-related text from the manuscript and let us know how you would like to update your Funding Statement. Currently, your Funding Statement reads as follows: [The authors received no specific funding for this work.]

Additionally, because some of your funding information pertains to [commercial funding//patents], we ask you to provide an updated Competing Interests statement, declaring all sources of commercial funding.

In your Competing Interests statement, please confirm that your commercial funding does not alter your adherence to PLOS ONE Editorial policies and criteria by including the following statement: ""This does not alter our adherence to PLOS ONE policies on sharing data and materials.” as detailed online in our guide for authors  http://journals.plos.org/plosone/s/competing-interests.  If this statement is not true and your adherence to PLOS policies on sharing data and materials is altered, please explain how.

Please include the updated Competing Interests Statement and Funding Statement in your cover letter. We will change the online submission form on your behalf.

3. When completing the data availability statement of the submission form, you indicated that you will make your data available on acceptance. We strongly recommend all authors decide on a data sharing plan before acceptance, as the process can be lengthy and hold up publication timelines. Please note that, though access restrictions are acceptable now, your entire data will need to be made freely accessible if your manuscript is accepted for publication. This policy applies to all data except where public deposition would breach compliance with the protocol approved by your research ethics board. If you are unable to adhere to our open data policy, please kindly revise your statement to explain your reasoning and we will seek the editor's input on an exemption. Please be assured that, once you have provided your new statement, the assessment of your exemption will not hold up the peer review process.

4. Please ensure that you include a title page within your main document. You should list the complete name of all authors and all affiliations as per our author instructions and clearly indicate the corresponding author.

5. Please include captions for your Supporting Information files at the end of your manuscript, and update any in-text citations to match accordingly. Please see our Supporting Information guidelines for more information: http://journals.plos.org/plosone/s/supporting-information.

6. If the reviewer comments include a recommendation to cite specific previously published works, please review and evaluate these publications to determine whether they are relevant and should be cited. There is no requirement to cite these works unless the editor has indicated otherwise.

Additional Editor Comments:

Dear Authors,

I appreciate your work concerning the Agrobacterium tumefaciens-mediated transformation of two fungal strains with fluorescent protein-encoding genes to elucidate the response on the host.

Tanacetum cinerariifolium (pyrethrum) through molecular visualization. Are these fluorescently labeled strains of both D. tanaceti and S. tanaceti natural isolates or registered fungal accessions?

Are these transformed strains co-infection shows same virulency as that of wild stains and whether can be used using cryopreservatives and cryoproectants for long term?

I have additional suggestions and comments:

Please mention the species like this Tanacetum cinerariifolium (Trevis.) Sch.Bip. when you introduce first time in the beginning of the text. Which type of a Agrobacterium tumefaciens strain was used for transformation? in some places the genus species Stagonosporopsis tanaceti was not italicized? Please italicize it.

What about the host specificity of these genetically modified strains or wild strains?

Are there any naturally resistant lines of Tanacetum cinerariifolium, or does any study show the natural resistance found against any of these fungal pathogens?

After sequencing the strains and confirming the T DNA using BLASTN, have you tried to find out the number of integrated copies of the T DNA? If so, how many copies of the T-DNA were found?

What kinds of genes were predicted using AUGUSTUS with the Botrytis cinerea reference? Have you predicted the fluorescent-related proteins alone or any more candidate genes and their proteins?

If so, could you please list them as a table with candidate gene IDs?

In the method section, the Detached Leaves Assay—D. tanaceti transformed strains analysis and the Detached Leaves Assay—S. tanaceti transformed strains analysis. almost follows the same type of analysis; could you please combine them to make it precise?

You have mentioned, "Microscopically, no apparent differences in fluorescence intensity were observed among the four S. tanaceti transformant strains." In contrast, slight variations were noted among D. tanaceti transformant strains." Any in planta transformed plant tissues represent the transformation of both these fungi, because you have mentioned co-infection of transformed D. tanaceti and S. tanaceti? Is there any difference between wild and transformed types of fungus in their activity and infection on the host tissues?

Please address the comments of reviewers and mine and revise the manuscript very precisely. One of the reviewers has rejected your manuscript; however, based on the revisions and responses, your manuscript may be considered further.

Reviewers' comments:

Reviewer's Responses to Questions

**Comments to the Author**

1. Is the manuscript technically sound, and do the data support the conclusions?

Reviewer #1: Yes

Reviewer #2: Partly

Reviewer #3: Yes

Reviewer #4: Partly

Reviewer #5: Yes

Reviewer #6: No

Reviewer #7: Yes

Reviewer #8: Yes

Reviewer #9: Partly

Reviewer #10: Yes

**********

2. Has the statistical analysis been performed appropriately and rigorously?

Reviewer #1: Yes

Reviewer #2: Yes

Reviewer #3: Yes

Reviewer #4: No

Reviewer #5: Yes

Reviewer #6: Yes

Reviewer #7: Yes

Reviewer #8: Yes

Reviewer #9: Yes

Reviewer #10: Yes

**********

3. Have the authors made all data underlying the findings in their manuscript fully available?

The PLOS Data policy

Reviewer #1: Yes

Reviewer #2: Yes

Reviewer #3: Yes

Reviewer #4: Yes

Reviewer #5: Yes

Reviewer #6: No

Reviewer #7: Yes

Reviewer #8: Yes

Reviewer #9: Yes

Reviewer #10: Yes

**********

4. Is the manuscript presented in an intelligible fashion and written in standard English?

Reviewer #1: Yes

Reviewer #2: Yes

Reviewer #3: Yes

Reviewer #4: Yes

Reviewer #5: Yes

Reviewer #6: Yes

Reviewer #7: Yes

Reviewer #8: Yes

Reviewer #9: Yes

Reviewer #10: Yes

**********

Reviewer #1: The following points detail the major concerns and necessary revisions for the manuscript. Addressing these issues is essential for validating the scientific rigor and clarity of the study.

1. Pathogen biomass and colonization dynamics should be validated using more precise techniques such as quantitative PCR (qPCR) or RT-qPCR. This is crucial to provide a definitive quantification of fungal load that complements the qualitative fluorescence data.

2. The authors should use clearer, high-contrast arrows (e.g., bright white or yellow) to ensure visibility against the background. Furthermore, the figure legends must be expanded to provide more descriptive explanations of these markers.

3. Since natural plant autofluorescence can often overlap or be confused with signals from fluorescent proteins, providing these controls is vital to confirm the specificity and accuracy of the pathogen signals.

4. There is a discrepancy in the captioning of Figure 7. While both panels A and B are presented in the figure, the sub-caption/legend only provides an explanation for panel A.

5. For the sake of clarity and scientific consistency, the authors must adopt a single unit of measurement throughout the manuscript. Please, standardize all time points to either HAI or DAI. both "Days After Inoculation" (DAI) and "Hours After Inoculation" (HAI) are used interchangeably (e.g., 24, 48, and 72 hours vs. 1, 2, and 3 days).

Reviewer #2: Dear editor,

This manuscript presents technically valuable work demonstrating the successful fluorescent transformation of Didymella tanaceti and Stagonosporopsis tanaceti and their visualization within leaf tissues of Tanacetum cinerariifolium. The development of stable fluorescent transformants is an important methodological contribution.

However, the current manuscript specially title and abstract overemphasize “pathogen interactions.” The study does not experimentally assess biological interactions between the two fungi beyond demonstrating their simultaneous presence in leaf tissues. In addition, disease biology and infection processes are only partially examined. Therefore, I recommend revising the title, abstract, and discussion to better reflect the main contribution of the study—namely, the successful transformation and in planta tracking of these pathogens—rather than implying that pathogen biology and interactions were elucidated.

Minor Comments

• Line 74: Revise “isolate UOM ST2 were” to “isolate UOM ST2 was”.

• Line 90: Add a comma before “respectively.”

• Line 97: Write Agrobacterium in italic format; keep the remaining words in regular font.

• Lines 108–111: Please rewrite the sentence for clarity.

• Line 128: Write the full genus name at the beginning of the sentence.

• Lines 214 and 235: Remove the full stop at the end of the sentence.

• Lines 217 and 238: Use either “60%” or “60 percent,” not both.

• Line 246: Write the full genus name at the beginning of the sentence.

Overall, the study is methodologically sound, but the framing should be aligned more closely with the actual scope and findings.

Best regards.

Reviewer #3: In general the manuscript was well written and the authors did a credible job in accomplishing their goal and I believe the results will be of interest to the readers. However there are some revisions required. Although details of methodology must be included but if the same procedure was repeated again and again it became cumbersome. Would it be possible to rephrase it as just for instance "as previously described "?

On the other hand other details should be included. Why some plates were sealed whereas others were unsealed? What determined when the cultures were incubated in light, user darkness or under light/dark 12 hours duration. I have problem with the measurement of colony diameter. It was stated that it was measured along the same line but where? I assume it was through the center of the plate. Normally, measurements are done along two straight lines perpendicular to each other through the center of the plate to account for any irregularities in the

colony outline. However. If the colonies were perfectly smooth and round I suppose one line should be sufficient.

Reviewer #4: Comment1

The manuscript « Elucidating pathogen interactions in Tanacetum cinerariifolium (pyrethrum) using fluorescently labelled Didymella tanaceti and Stagonosporopsis tanaceti» describes the creation of fluorescently tagged Didymella tanaceti and Stagonosporopsis tanaceti strains for pyrethrum infection studies. While the technological success of producing these tagged pathogens is impressive, the work has numerous important methodological flaws that restrict its usefulness to plant pathology research.

Comment 2

This paper has a mismatch between its declared aims and actual findings. The title presented insights into pathogen interactions in plants, however the data show only co-localization and no evidence of biological interactions, such as competitive assays, antagonistic effects, or quantitative interference analysis (evidence of competitive, synergistic, or antagonistic effects). The work evolved into a techniques validation publication (70% concentrate on transformation protocols) rather than a biological exploration of the two pathogens interaction.

I recommend either:

1. Replace the current title with one that accurately reflects the study's main results.

2. Retain the current title but add essential experiments to substantiate the claimed pathogen interactions. A competitive fitness assays comparing single vs. co-infection scenarios with quantitative disease severity measurements is recommended. A resource competition experiments measuring nutrient depletion effects when pathogens occupy the same tissue as well as plant defense response comparisons between single and co-infections using gene expression analysis, must be added.

Comment 3

I have several reservations about the organization of the manuscript. Some findings were hidden in the discussion section: Lines 558-567 (epidermal penetration), 570-581 (infection intervals), 583-596 (co-infection observations), and 598-618 (autofluorescence characterisation) include primary data that should be in findings rather than discussion. The discussion section is long and should be reduced or shifted to additional material, particularly thorough technical validation details that do not increase biological knowledge.

Comment 4

The manuscript has certain key methodological deficiencies: In terms of statistical analysis and experimental design, Line 155 lacks software specifications, alpha values, and ANOVA assumption testing. Because of the small sample numbers used in detached leaf tests, power analysis must be justified. The paper does not give any information on the replication structure (Biological and technical replicates). Furthermore, the detached leaf test technique is insufficient to validate biological equivalency due to the absence of quantitative disease severity score and no entire plant validation beyond detached leaves especially in the context of pathogens interaction in-planta.

Comment 5

There are various limits to genome analysis. The assembly quality measures are lacking, as they lack N50 values and contig counts. If feasible, I would consider comparing the BUSCO data to other Didymellaceae genomes. Genome statistics (lines 322-329) should be tabulated rather than included in the text.

Comment 6

Figure 7 does not appropriately display the co-infection data since it only shows microscopic cross-sections without the necessary macroscopic context. The figure should include full leaf pictures (e.g., Figs. 5 and 6) that demonstrate lesion progression, and sampling points where sections were collected. Without this context, we cannot determine if co-infection has synergistic, antagonistic, or additive disease consequences, or how pathogen colonization patterns relate to symptom expression.

Comment 7

Given the experimental methodology, the result on direct epidermal penetration (line 558) seems dubious. Within hours, detached leaves lose their stomatal function, which may make epidermal penetration the only viable route. To confirm biological results about natural infection methods, this methodological bias has to be validated using intact plants and functioning stomata.

Comment 8

Instead of presenting incomplete interaction studies, this study would be greatly boosted if it were repositioned as the basis for future biological research. Expand the biological characterization with these verified techniques and transfer comprehensive transformation methods to supplemental material. Although the fluorescent strains are useful research tools, they require significant refinement in their biological application before they can be published.

Reviewer #5: The article is thorough, well-organized, and clearly presents data on transformation efficiency, fluorescence validation, morphological assessment, molecular characterization, and pathogenicity comparisons. The incorporation of microscopy, quantitative fluorescence analysis (CTCF), genome assembly statistics, BUSCO completeness assessments, and T-DNA insertion mapping effectively supports the study's findings. Nonetheless, certain areas would benefit from clarification, consistency corrections, grammatical improvements, and enhanced data presentation to further strengthen scientific clarity and readability.

• Confidence intervals or error bars (SD/SE) should be specified in figure legends.

• The p-value range reported for D. tanaceti (p = 0.02 – 0.0002) is unclear. Exact p-values should be reported for each comparison.

• You mentioned that 15 ROIs per transformant, need to include the number of biological replicates

• Some paragraphs are overly detailed, particularly: T-DNA rearrangements discussion, autofluorescence explanation and literature comparisons.

• Improve explanation of findings and discussion

• Include proper units and legends

• Condense repetitive statements and tighten literature comparison sections.

• Briefly reinforce statistical validation if performed.

Reviewer #6: The authors wrote this manuscript well for their construction of fluorescent fungal pathogens of pyrethrum and the preliminary observation of the fluorescent images of the derived transformants in planta including single or co-inoculation. The procedures for the construction of fluorescent strains are described in detail and really attain the goal. If the quality of the image can be improved, that will be better or explain the present image quality. Elucidating pathogen interactions in planta by using the resulting fluorescent strains requires more experiments to show their interactions. Symptom caused by co-inoculation of these two fungi was not shown, whether they succeed in causing lesion and the extent of infection were not shown in this study.

Line 30: Specify "knowledge gap exists regarding the infection biology of D. tanaceti" in pyrethrum.

Line 75: single spore isolation.

Lines 129-130: note the reason for using hyphae-tip isolation instead of single spore isolation for S. tanaceti.

Line 148: ROI (w=1.96, h=1.96) in hyphae sections. Note that the ROI stand for.

Lines 225-234: need rewording for clearer description.

Line 256: using Fiji?

Omit figure legends in the results section.

Line 481: UOM DT5 also has partial T-DNA insertion. This strain has single T-DNA insertion. So, make sure the description is correct or restate the situation.

Fig. 6: wrong pictures on (C) and (D), suppose to be exchanged.

Reviewer #7: The manuscript is experimentally properly designed. The research is organized straightforwardly to investigate the transformed strains with respect to pathogenicity, morphology, and fluorescence emission. The manuscript is well written, and the statistical analyses meet the purpose of the research.

Reviewer #8: The study describes an useful tool/technique to study the fungal pathogens (fungal pathogens Didymella tanaceti and Stagonosporopsis tanaceti) infection of the host Pyrethrum (Tanacetum cinerariifolium) which is an economically important crop for its use as in insecticides. Using the Agrobacterium mediated transformation of these fungi the authors have generated fluorescent label strains that can help gain better insights into the infection process. However, it would have been more convincing to see the gene expression analysis of the genes affected by/flanking the insertions as a result of the T-DNA insertion. Also the results have 3-4% genes missing from analysis. T-DNA transformations can also lead to integration and excision of the T-DNA from sites if it is not stable. In which case it may also cause disruption at these sites which may be regulatory or coding regions. In that light it is not too convincing to have >200 genes missing from the sequencing data analysis.

There are minor formatting errors, missed informations, and formatting incongruity in the references and figure legends that can be easily taken care of.

My detailed comments are attached with the pdf file of the review manuscripts. At this point I would like the authors to address those concerns and resubmit.

Reviewer #9: Dear Authors,

This manuscript presents a technically valuable output by developing fluorescently labelled strains of Didymella tanaceti and Stagonosporopsis tanaceti. The successful transformation and characterisation of these strains represent a methodological advance that will benefit the pyrethrum community. However, the manuscript has some major issues regarding the experimental design, data interpretation, and scientific writing that require revision before it meets the requirements for publication.

The comment are list in the attached files

Reviewer #10: The study is technically sound, well designed, and addresses an important gap in understanding the biology of these pathogens. The combination of Agrobacterium-mediated transformation and whole-genome sequencing to confirm T-DNA insertion sites is a strong aspect of the work. The data support the conclusions and the manuscript is clearly written and logically organized. The microscopy, CTCF quantification, and molecular characterization are well presented, and the infection and co-infection assays provide useful insights into colonisation patterns.

I recommend acceptance after minor revision. A few points stated in the attached file could be clarified.

Overall, this is a solid and useful contribution to fungal plant pathology, and the fluorescent strains generated here will be valuable tools for future studies.

**********

what does this mean?). If published, this will include your full peer review and any attached files.

If you choose “no”, your identity will remain anonymous but your review may still be made public.

**Do you want your identity to be public for this peer review?**  For information about this choice, including consent withdrawal, please see our Privacy Policy

Reviewer #1: **Yes:**  Büşra ALBAYRAK TURGUT

Reviewer #2: **Yes:**  Leila Ebrahimi

Reviewer #3: No

Reviewer #4: **Yes:**  Manel Chaouachi

Reviewer #5: No

Reviewer #6: No

Reviewer #7: **Yes:**  Loukas Kanetis

Reviewer #8: No

Reviewer #9: No

Reviewer #10: No

**********

[NOTE: If reviewer comments were submitted as an attachment file, they will be attached to this email and accessible via the submission site. Please log into your account, locate the manuscript record, and check for the action link "View Attachments". If this link does not appear, there are no attachment files.]

To ensure your figures meet our technical requirements, please review our figure guidelines: https://journals.plos.org/plosone/s/figures

You may also use PLOS’s free figure tool, NAAS, to help you prepare publication quality figures: https://journals.plos.org/plosone/s/figures#loc-tools-for-figure-preparation.

NAAS will assess whether your figures meet our technical requirements by comparing each figure against our figure specifications.

Attachment Submitted filename: Review report.docx

Attachment Submitted filename: PONE-D-25-58448_reviewer.pdf

Attachment Submitted filename: Review_PONE-D-25-58448.pdf

Attachment Submitted filename: Manuscript_VC_2025 (2).docx

# Author comment: Author response to Decision Letter 1

**DOI:** 10.1371/journal.pone.0355715.r002

6 May 2026

This is also provided in a Table format in the file uploaded as Response to Reviewers

Dear reviewers,

We thank the reviewers and editor for their thorough evaluation of our manuscript and for their constructive comments. These suggestions have significantly improved the clarity, structure, and focus of the study. All comments have been carefully considered and addressed, as detailed below:

“Are these fluorescently labelled strains of both D. tanaceti and S. tanaceti natural isolates or registered fungal accessions?”

We thank the reviewer for this question. Stagonosporopsis tanaceti isolate UOM ST2 is a natural isolate, whereas Didymella tanaceti strain BRIP 61988 is a registered accession. The corresponding reference describing BRIP 61988 has been included at its first mention in the manuscript (Page 4, line 126).

“Which type of a Agrobacterium tumefaciens strain was used for transformation? in some places the genus species Stagonosporopsis tanaceti was not italicized? Please italicize it”

The Agrobacterium tumefaciens strain used for transformation has now been specified in the main text (Page 6, line 180). In addition, all occurrences of Stagonosporopsis tanaceti have been carefully revised and are now consistently italicised throughout the manuscript.

“Are there any naturally resistant lines of Tanacetum cinerariifolium, or does any study show the natural resistance found against any of these fungal pathogens?”

To our knowledge, there are currently no well-documented naturally resistant lines of Tanacetum cinerariifolium against these pathogens. Pyrethrum breeding programmes have historically focused on traits such as pyrethrin yield rather than disease resistance.

“The manuscript overemphasises pathogen interactions. The title, abstract, and discussion should better reflect the main contribution”

We thank the reviewer for this comment. The manuscript has been substantially revised to better reflect its primary focus. The title, abstract, and discussion have been reframed to emphasise the development and characterisation of fluorescent transformants and their application for visualisation in planta, rather than suggesting a comprehensive analysis of pathogen interactions. Statements implying detailed interaction analysis have been removed or moderated, and the infection observations are now presented as an initial descriptive framework.

“After sequencing the strains and confirming the T DNA using BLASTN, have you tried to find out the number of integrated copies of the T DNA? If so, how many copies of the T-DNA were found?”

We thank the reviewer for this comment. The number of T-DNA copies was assessed using BLAST-based analysis against the assembled genomes, as described in the Methods. Based on this approach, all transformants analysed contained a single complete T-DNA insertion. This has now been clarified in the Results section (Page 17, Lines 598-602)

“What kinds of genes were predicted using AUGUSTUS with the Botrytis cinerea reference? Have you predicted the fluorescent-related proteins alone or any more candidate genes and their proteins? If so, could you please list them as a table with candidate gene IDs”

We thank the reviewer for this comment. Gene prediction using AUGUSTUS was performed to identify gene models in regions surrounding T-DNA insertion sites. As AUGUSTUS output in Geneious did not provide functional annotation, predicted genes were further analysed using BLAST to infer putative functions. Analyses were restricted to genes associated with insertion sites rather than genome-wide predictions. A supplementary table has been added summarising these genes, including AUGUSTUS gene IDs and BLAST-based annotations.

“Pathogen biomass and colonization dynamics should be validated using more precise techniques such as quantitative PCR (qPCR) or RT-qPCR. This is crucial to provide a definitive quantification of fungal load that complements the qualitative fluorescence data”

We thank the reviewer for this suggestion. We agree that quantitative approaches such as qPCR or RT-qPCR would provide valuable insights into pathogen biomass and colonisation dynamics. However, these analyses were beyond the scope of the present study, which focuses on the generation and characterisation of fluorescent transformants and qualitative description of infection processes. A statement has been added to the Discussion highlighting this as an important direction for future work (Page 27, Line 1179)

Co-infection and interaction claims We thank the reviewer for these comments. All sections related to pathogen-pathogen interactions and co-infection analysis have been removed from the manuscript to avoid overinterpretation. The study has been refocused to emphasise the development of fluorescent strains and their application for visualisation. Observations of co-localisation are now presented only as preliminary and descriptive, demonstrating feasibility rather than biological interaction.

“Some findings were hidden in the discussion section: Lines 558-567 (epidermal penetration), 570-581 (infection intervals), 583-596 (co-infection observations), and 598-618 (autofluorescence characterisation) include primary data that should be in findings rather than discussion”

We thank the reviewer for this important observation. Sections previously containing primary observations in the Discussion have been revised. Relevant findings (e.g., infection timing, co-localisation observations, and autofluorescence characterisation) have been moved or appropriately integrated into the Results section. The Discussion has been restructured to focus on biological interpretation rather than presentation of results.

“The discussion section is long and should be reduced or shifted to additional material, particularly thorough technical validation details that do not increase biological knowledge”

We thank the reviewer for this suggestion. The Discussion has been substantially shortened and restructured to remove excessive technical detail and improve focus on biological interpretation.

“The manuscript has certain key methodological deficiencies: In terms of statistical analysis and experimental design, Line 155 lacks software specifications, alpha values, and ANOVA assumption testing. Because of the small sample numbers used in detached leaf tests, power analysis must be justified”

We thank the reviewer for this comment. Statistical methods have been clarified, including software specifications, significance threshold (α = 0.05), and assumption testing. The detached leaf assay has been explicitly described as a qualitative, exploratory experiment rather than a fully powered quantitative analysis, and now this has been clarified in the Methods.

“The paper does not give any information on the replication structure (Biological and technical replicates)”

We thank the reviewer for this comment. The replication structure has now been clarified in the Methods section. Each leaflet was treated as an independent biological replicate, and fluorescence measurements consisted of technical replication (multiple ROIs) within a single biological sample.

“Furthermore, the detached leaf test technique is insufficient to validate biological equivalency due to the absence of quantitative disease severity score and no entire plant validation beyond detached leaves especially in the context of pathogens interaction in-planta”

We thank the reviewer for this comment. The manuscript has been revised to clarify that the detached leaf assay was used as a controlled, qualitative system to describe early infection processes rather than to assess quantitative disease severity or full biological equivalence. The limitations of this approach and the need for future whole-plant studies have been explicitly acknowledged in the Discussion.

“There are various limits to genome analysis. The assembly quality measures are lacking, as they lack N50 values and contig counts. If feasible, I would consider comparing the BUSCO data to other Didymellaceae genomes. Genome statistics (lines 322-329) should be tabulated rather than included in the text”

We thank the reviewer for this suggestion. Genome statistics, including assembly size, contig counts, and N50 values, have now been tabulated for clarity (Page 16). BUSCO results have been retained in the text. This improves presentation and facilitates comparison across strains.

“Given the experimental methodology, the result on direct epidermal penetration (line 558) seems dubious. Within hours, detached leaves lose their stomatal function, which may make epidermal penetration the only viable route. To confirm biological results about natural infection methods, this methodological bias has to be validated using intact plants and functioning stomata”

We thank the reviewer for this insightful comment. We acknowledge that detached leaf assays may influence stomatal function. The Discussion has been revised to clarify that observations of direct epidermal penetration are limited to the experimental conditions used (Page 26, Line 1061) and do not exclude alternative infection routes.

“This study would be greatly boosted if it were repositioned as the basis for future biological research. Expand the biological characterization with these verified techniques and transfer comprehensive transformation methods to supplemental material. Although the fluorescent strains are useful research tools, they require significant refinement in their biological application before they can be published”

We thank the reviewer for this perspective. The manuscript has been refocused to emphasise the development and validation of fluorescent transformants, while presenting infection observations as an initial descriptive framework to support future biological studies.

Figure captions: request to remove from main text

Figure captions have been retained in the main manuscript text in accordance with PLOS ONE guidelines, which state that “figure captions should be placed in the manuscript text in read order, immediately following the paragraph where the figure is first cited.” Supplementary figures and tables have been placed after the references, as recommended.

Supplementary materials and media composition:

We thank the reviewer for this comment. The full composition of all media has been provided in the Supplementary Materials, including ingredient concentrations and preparation steps. Manufacturer details have been included at first mention in the manuscript.

“Why is D. tanaceti the more important?” We thank the reviewer for this comment. Context of the disease now added as part of the introduction (lines 60-70)

This reads more like a review on fluorescent proteins than necessary background for this study. You can compress it a little bit." We thank the reviewer for this comment. Introductory section on fluorescent proteins has now been compressed (lines 73-114)

“what type of light? shelf, BOD" We thank the reviewer for this comment. The type of light source has now been clarified by specifying the growth chamber used for incubation in the Methods section (lines 144-146)

"Clarify whether kanR remains in the final constructs or was replaced".

We thank the reviewer for this comment. Now clarified (line 153)

Write in standard fromat (Optical density 600) We thank the reviewer for this comment, standard format now added (line 181)

“Briefly justify why different purification methods were used to” We thank the reviewer for this comment, detail now added in lines 214-215

clarify ROI unit. We thank the reviewer for this comment. ROI unit is now clarified (line 240-241)

Size of agar plug subcultures We thank the reviewer for this comment. Agar plug size now clarified (Line 258)

“What was the DNA template concentration” We thank the reviewer for this comment. DNA concentrations were measured using a Qubit fluorometer. As no PCR or reaction-based amplification steps were performed prior to sequencing, no template DNA concentration per reaction was applicable.

Replace integration for RETENTION. Up to now, fluorescence retention demonstrates stable expression, but it has not necessarily proven genomic integration. We thank the reviewer for this comment. Replaced with correct wording (line 451)

“Please either provide exact p-values per comparison or report overall ANOVA p-value and Tukey grouping" We thank the reviewer for this comment. Exact p values now provided as part of the results (Lines 488-493)

How many biological replicates were used to assess colony growth rate?

Was radial growth measured quantitatively or visually? We thank the reviewer for this comment. Number of replicates is specified in methods (line 257). Radial growth was measured quantitatively data provided in supplementary table

Why are transformed assemblies slightly smaller than the estimated genome size? We thank the reviewer for this comment. This has now been added as part of the discussion (Lines 895-898)

Were lesion sizes measured quantitatively?

Was disease severity statistically compared? We thank the reviewer for this comment. Lesion size and disease severity were not quantified in this study, as the primary objective was to assess the feasibility of using fluorescently labelled strains to visualise infection and colonisation processes. The observations presented are therefore qualitative and intended to provide an initial description of infection dynamics. Quantitative assessment of disease severity and lesion development represents an important direction for future work.

Was this based on replicated observations? If yes, specify the number of replicates We thank the reviewer for this comment. Number of replicates now added to manuscript (line 695)

Was any autofluorescence control performed (mock-inoculated leaves under DsRed filter)?

Were exposure settings standardised between wild type and transformant?

I am asking about false positives due to autofluorescence We thank the reviewer for this comment. Yes, autofluorescence controls were considered during image acquisition. Mock-inoculated leaf tissues were examined under the DsRed and YFP filter sets to assess background fluorescence. Exposure settings were standardised across all images (1 s exposure) to ensure comparability between wild-type and transformant samples.

To minimise false positives, fluorescence signals were cross-checked between channels. Signals observed under the DsRed filter were also examined under the YFP filter; persistent fluorescence across both channels was interpreted as plant autofluorescence, whereas signals restricted to a single channel were attributed to the fluorescent protein. The same approach was applied for Didymella tanaceti transformants.

While representative images of these controls were not retained, this comparative approach was consistently used to distinguish true fluorescence from background autofluorescence

In the discussion section: Can you state explicitly what was previously unknown about D. tanaceti (entry mode? colonisation timing? tissue specificity?) to sharpen the gap further? We thank the reviewer for this comment. Specific gap now added (line 829-831)

Could copy number differences explain intensity variation? We thank the reviewer for this comment. Differences in fluorescence intensity are unlikely to be explained by variation in T-DNA copy number, as all selected transformants were confirmed to contain a single insertion. However, variation in fluorescence intensity may be influenced by the genomic location of the T-DNA insertion

Do you have preliminary evidence of systemic movement? Or is this purely prospective? We thank the reviewer for this comment. At this stage, there is no direct evidence supporting systemic movement of the pathogen in this study. The suggestion is therefore prospective and based on observations reported in other plant–pathogen systems.

Either use DAI or HAI We appreciate the reviewer’s comment regarding unit standardisation. In this study, hours after inoculation (HAI) were used to describe early infection events observed microscopically (e.g. spore germination, germ tube emergence, and penetration), which occur over short timeframes. In contrast, days after inoculation (DAI) were used

Attachment Submitted filename: Response to reviewers.docx

# Peer review: Decision Letter 1

**DOI:** 10.1371/journal.pone.0355715.r003

**Authors:** Karthikeyan Thiyagarajan

5 Jun 2026

Dear Dr.  Carrillo Portillo

Thank you for submitting your manuscript to PLOS ONE. After careful consideration, we feel that it has merit but does not fully meet PLOS ONE’s publication criteria as it currently stands. Therefore, we invite you to submit a revised version of the manuscript that addresses the points raised during the review process.

Please submit your revised manuscript by Jul 20 2026 11:59PM. If you will need more time than this to complete your revisions, please reply to this message or contact the journal office at plosone@plos.org. When you're ready to submit your revision, log on to https://www.editorialmanager.com/pone/ and select the 'Submissions Needing Revision' folder to locate your manuscript file.

- A letter that responds to each point raised by the academic editor and reviewer(s). You should upload this letter as a separate file labeled 'Response to Reviewers'.
- A marked-up copy of your manuscript that highlights changes made to the original version. You should upload this as a separate file labeled 'Revised Manuscript with Track Changes'.
- An unmarked version of your revised paper without tracked changes. You should upload this as a separate file labeled 'Manuscript'.

If you would like to make changes to your financial disclosure, please include your updated statement in your cover letter. Guidelines for resubmitting your figure files are available below the reviewer comments at the end of this letter.

If applicable, we recommend that you deposit your laboratory protocols in protocols.io to enhance the reproducibility of your results. Protocols.io assigns your protocol its own identifier (DOI) so that it can be cited independently in the future. For instructions see: https://journals.plos.org/plosone/s/submission-guidelines#loc-laboratory-protocols. Additionally, PLOS ONE offers an option for publishing peer-reviewed Lab Protocol articles, which describe protocols hosted on protocols.io. Read more information on sharing protocols at https://plos.org/protocols?utm_medium=editorial-email&utm_source=authorletters&utm_campaign=protocols.

As the corresponding author, your ORCID iD is verified in the submission system and will appear in the published article. PLOS supports the use of ORCID, and we encourage all coauthors to register for an ORCID iD and use it as well. Please encourage your coauthors to verify their ORCID iD within the submission system before final acceptance, as unverified ORCID iDs will not appear in the published article. *Only*  the individual author can complete the verification step; PLOS staff *cannot*  verify ORCID iDs on behalf of authors.

We look forward to receiving your revised manuscript.

Kind regards,

Karthikeyan Thiyagarajan, PhD

Academic Editor

PLOS One

Journal Requirements:

If the reviewer comments include a recommendation to cite specific previously published works, please review and evaluate these publications to determine whether they are relevant and should be cited. There is no requirement to cite these works unless the editor has indicated otherwise.

Please review your reference list to ensure that it is complete and correct. If you have cited papers that have been retracted, please include the rationale for doing so in the manuscript text, or remove these references and replace them with relevant current references. Any changes to the reference list should be mentioned in the rebuttal letter that accompanies your revised manuscript. If you need to cite a retracted article, indicate the article’s retracted status in the References list and also include a citation and full reference for the retraction notice.

Additional Editor Comments:

Dear Authors,

I appreciate your revisions and responses; however, there is a need for a minor revision.

There are some typos; please check the standard convention for "in planta."

Please check the reviewers' comments, respond to them, and revise the manuscript accordingly.

Reviewers' comments:

Reviewer's Responses to Questions

**Comments to the Author**

Reviewer #1: All comments have been addressed

Reviewer #3: (No Response)

Reviewer #4: All comments have been addressed

Reviewer #6: All comments have been addressed

**********

2. Is the manuscript technically sound, and do the data support the conclusions?

Reviewer #1: Yes

Reviewer #3: Yes

Reviewer #4: Yes

Reviewer #6: Partly

**********

3. Has the statistical analysis been performed appropriately and rigorously?

Reviewer #1: Yes

Reviewer #3: Yes

Reviewer #4: Yes

Reviewer #6: No

**********

4. Have the authors made all data underlying the findings in their manuscript fully available?

The PLOS Data policy

Reviewer #1: Yes

Reviewer #3: Yes

Reviewer #4: Yes

Reviewer #6: No

**********

5. Is the manuscript presented in an intelligible fashion and written in standard English?

Reviewer #1: Yes

Reviewer #3: Yes

Reviewer #4: Yes

Reviewer #6: No

**********

Reviewer #1: I have reviewed the revised manuscript. The author has addressed most of the corrections I suggested, and the manuscript has improved significantly. I recommend that the manuscript be accepted this version.

Reviewer #3: In general the manuscript was written prepared and presented. Here are some comments.

Line 18 insert a period after losses and start a new sentence with However.

Line 60 Insert a period after tissues and start a new sentence with However.

Line 100 what was the pore size of the millipore? Express HAI first as Hours after infection. Why were the cultures sometimes incubated under light and sometimes under light/darkness.

Line 185 How many plates were measured to get the average colony diameter.

Line 502 Appropriate reference should be cited here. You wondered if the pathogen could enter through the stomata did you try to infect the leaves on the lower epidermis where most stomata are located?

Reviewer #4: All comments raised by the reviewers have been carefully considered by the authors, and the corresponding corrections and revisions have been fully implemented.

Reviewer #6: 1. The quality of figure 2 is quite poor. The resolution should be improved, also check the format required for formal publication. Line 163-166, add the fluorescence quantification method and the information of dichroic filters. The emission filters are long-pass or band-pass ones?

2. Lines 336-339. Why was spore size measurement only for D. tanaceti but not for S. tanaceti? It should be determined if there is statistical difference in spore size between wild-type and transformed strains. Besides, the sporulation speed and amount between wild-type and transformed strains are better to be compared since they often affect fungal virulence.

3. Since the transformed strains are intended to be used in pathological study, the comparable virulence of wild-type and transformed strained strains on hosts is required. The analysis in fungal biomass and plant lesion developments would help.

4. Lacking the medium formulas used in transformant construction.

Line 126, Liquid cultures of Agrobacterium strain EHA105 “in LB broth. Add LB medium formula.

Line 129, Add the formula of induction medium.

**********

what does this mean?). If published, this will include your full peer review and any attached files.

If you choose “no”, your identity will remain anonymous but your review may still be made public.

**Do you want your identity to be public for this peer review?**  For information about this choice, including consent withdrawal, please see our Privacy Policy

Reviewer #1: **Yes:**  Büşra ALBAYRAK TURGUT

Reviewer #3: No

Reviewer #4: No

Reviewer #6: No

**********

[NOTE: If reviewer comments were submitted as an attachment file, they will be attached to this email and accessible via the submission site. Please log into your account, locate the manuscript record, and check for the action link "View Attachments". If this link does not appear, there are no attachment files.]

To ensure your figures meet our technical requirements, please review our figure guidelines: https://journals.plos.org/plosone/s/figures

You may also use PLOS’s free figure tool, NAAS, to help you prepare publication quality figures: https://journals.plos.org/plosone/s/figures#loc-tools-for-figure-preparation.

NAAS will assess whether your figures meet our technical requirements by comparing each figure against our figure specifications.

# Author comment: Author response to Decision Letter 2

**DOI:** 10.1371/journal.pone.0355715.r004

27 Jun 2026

Dear reviewers,

We thank the reviewers and editor for their thorough evaluation of our manuscript and for their constructive comments. These suggestions have further improved the clarity, structure, and focus of the study. All comments have been carefully considered and addressed, as detailed below:

Comment Response

1. Line 18 insert a period after losses and start a new sentence with However.

Thank you for the comment on sentence structure. The error has been amended

2. Line 60 Insert a period after tissues and start a new sentence with However.

Thank you for the comment. The error was in line 61, and is now amended

3. Line 100 what was the pore size of the millipore? Express HAI first as Hours after infection. Why were the cultures sometimes incubated under light and sometimes under light/darkness.

The pore size of the Miracloth used for filtration has now been added to the Materials and Methods section (22-25 µm).

Regarding the use of the term "inoculation", we have retained this terminology throughout the manuscript. Inoculation refers to the introduction of the pathogen onto the host, whereas infection refers to the subsequent establishment and colonisation of host tissues. As infection is not necessarily confirmed at the time of pathogen application, we consider "inoculation" to be the most appropriate term in this context.

Thank you for picking this up. The incubation condition reported in line 144 has been corrected to darkness. This incubation step was conducted in a different laboratory associated with work involving genetically modified microorganisms at the university. Consequently, the incubation conditions at this stage differed from those used in subsequent experiments.

4. Line 185 How many plates were measured to get the average colony diameter.

Thank you very much for your comment. Average colony diameter was measured from three replicate plates for each isolate. This information is provided in the Materials and Methods section (Line 188).

5. Line 502 Appropriate reference should be cited here. You wondered if the pathogen could enter through the stomata did you try to infect the leaves on the lower epidermis where most stomata are located? Thank you for your comment. We believe there may be a discrepancy between the comment and the cited line number. Line 502 (now line 544) refers to the discussion of T-DNA integration sites and the putative function of the Rad5-family DNA repair protein, which is supported by references 52-54. We did find an error in Line 566:

“Glandular trichomes on the surface of pyrethrum leaves are known to exhibit intrinsic fluorescence across multiple wavelengths [68-70]. [71]”).

This has now been corrected. We would be very grateful if the reviewer could clarify the intended section if a different passage was being referred to.

We have clarified in the Discussion (Line 612) that potential penetration through stomata was not directly observed in this study and remains speculative. This has been identified as an area requiring further investigation.

6. The quality of figure 2 is quite poor. The resolution should be improved, also check the format required for formal publication. Thank you for your comment. Figure 2, as well as other figures has been reformatted and resubmitted in TIFF format at 300 dpi. The figure dimensions have also been adjusted to comply with the image size and resolution requirements specified by PLOS ONE for publication.

7. Line 163-166, add the fluorescence quantification method and the information of dichroic filters. The emission filters are long-pass or band-pass ones? Thank you for your comment: Fluorescent filters information is now added into the paragraph for completeness. Emission filters are band-pass. Fluorescent quantification description and formula is located from line 169 to 185.

8. Lines 336-339. Why was spore size measurement only for D. tanaceti but not for S. tanaceti? It should be determined if there is statistical difference in spore size between wild-type and transformed strains. Besides, the sporulation speed and amount between wild-type and transformed strains are better to be compared since they often affect fungal virulence. Thank you for this suggestion. Additional conidial measurements were conducted for both D. tanaceti and S. tanaceti isolates. Conidial length and width were measured for the wild-type and transformed strains, and statistical comparisons were performed using one-way ANOVA followed by pairwise comparisons of estimated marginal means. The Methods, Results, and Discussion sections have been updated accordingly. For S. tanaceti, conidial measurements were obtained from the wild type isolate UOM ST2 and the transformed isolate UOMSTC1R2, as these were the isolates sporulating at the time of assessment

Comparison of sporulation speed and sporulation quantity was not undertaken in the present study. Sporulation in S. tanaceti is normally difficult and inconsistent in non-transformed isolates. In this study, sporulation was very variable and inconsistent among both wild type and transformed isolates under the conditions tested, making reliable quantitative comparisons difficult.

9. Since the transformed strains are intended to be used in pathological study, the comparable virulence of wild-type and transformed strained strains on hosts is required. The analysis in fungal biomass and plant lesion developments would help. Thank you for this suggestion. We agree that quantitative assessments of virulence, including lesion development and fungal biomass measurements, would provide additional validation of the biological equivalence of transformed and parental strains. However, the primary objective of this study was the generation and characterisation of fluorescent transformants for future infection biology studies. During pathogenicity assays, transformed strains successfully infected host tissue and produced lesions comparable to those observed for the parental strains, with no obvious differences in symptom development. Furthermore, the manuscript already acknowledges the need for future whole-plant inoculation assays, quantitative disease assessments and qPCR-based biomass quantification to further investigate pathogen colonisation and disease development under physiological conditions.

10. Lacking the medium formulas used in transformant construction. Thank you for the feedback. Added formulas for Lysogeny Broth and Induction Media in the main text and further preparation is contained in Supplementary material

11. Line 126, Liquid cultures of Agrobacterium strain EHA105 “in LB broth. Add LB medium formula. Thank you for the feedback. Now added in text

12. Line 129, Add the formula of induction medium. Thank you for the feedback. Now added in text

We believe that these revisions have significantly improved the clarity, focus, and scientific rigour of the manuscript. We thank the reviewers again for dedicating their valuable time and detailed feedback, and hope that the revised version meets the expectations for publication.

Attachment Submitted filename: Response_to_reviewers_auresp_2.docx

# Editor report: Decision Letter 2

**DOI:** 10.1371/journal.pone.0355715.r005

**Authors:** Karthikeyan Thiyagarajan

20 Jul 2026

Thank you for submitting your manuscript to PLOS ONE. After careful consideration, we feel that it has merit but does not fully meet PLOS ONE’s publication criteria as it currently stands. Therefore, we invite you to submit a revised version of the manuscript that addresses the points raised during the review process.

Please submit your revised manuscript by Sep 03 2026 11:59PM. If you will need more time than this to complete your revisions, please reply to this message or contact the journal office at plosone@plos.org. When you're ready to submit your revision, log on to https://www.editorialmanager.com/pone/ and select the 'Submissions Needing Revision' folder to locate your manuscript file.

- A letter that responds to each point raised by the academic editor and reviewer(s). You should upload this letter as a separate file labeled 'Response to Reviewers'.
- A marked-up copy of your manuscript that highlights changes made to the original version. You should upload this as a separate file labeled 'Revised Manuscript with Track Changes'.
- An unmarked version of your revised paper without tracked changes. You should upload this as a separate file labeled 'Manuscript'.

If you would like to make changes to your financial disclosure, please include your updated statement in your cover letter. Guidelines for resubmitting your figure files are available below the reviewer comments at the end of this letter.

If applicable, we recommend that you deposit your laboratory protocols in protocols.io to enhance the reproducibility of your results. Protocols.io assigns your protocol its own identifier (DOI) so that it can be cited independently in the future. For instructions see: https://journals.plos.org/plosone/s/submission-guidelines#loc-laboratory-protocols. Additionally, PLOS ONE offers an option for publishing peer-reviewed Lab Protocol articles, which describe protocols hosted on protocols.io. Read more information on sharing protocols at https://plos.org/protocols?utm_medium=editorial-email&utm_source=authorletters&utm_campaign=protocols.

As the corresponding author, your ORCID iD is verified in the submission system and will appear in the published article. PLOS supports the use of ORCID, and we encourage all coauthors to register for an ORCID iD and use it as well. Please encourage your coauthors to verify their ORCID iD within the submission system before final acceptance, as unverified ORCID iDs will not appear in the published article. *Only*  the individual author can complete the verification step; PLOS staff *cannot*  verify ORCID iDs on behalf of authors.

We look forward to receiving your revised manuscript.

Kind regards,

Karthikeyan Thiyagarajan, PhD

Academic Editor

PLOS One

Journal Requirements:

If the reviewer comments include a recommendation to cite specific previously published works, please review and evaluate these publications to determine whether they are relevant and should be cited. There is no requirement to cite these works unless the editor has indicated otherwise.

Please review your reference list to ensure that it is complete and correct. If you have cited papers that have been retracted, please include the rationale for doing so in the manuscript text, or remove these references and replace them with relevant current references. Any changes to the reference list should be mentioned in the rebuttal letter that accompanies your revised manuscript. If you need to cite a retracted article, indicate the article’s retracted status in the References list and also include a citation and full reference for the retraction notice.

Additional Editor Comments:

Dear Authors,

I appreciate your revision and responses, however I suggest to do some minor corrections:

Transformant UOMSTC1R2 is mentioned in two ways: UOM STC1R2 and UOMSTC1R2. Please mention the correct one.

You have used the k-mer counting software Jellyfish v.2.3.1, and you have also mentioned the following: The estimated genome size for D. tanaceti isolates was 40.7 Mbp using Jellyfish. Here 'Jellyfish' means the software, doesn't it? So, please mention the software with an appropriate version throughout the manuscript.

Also in the introduction you have mentioned the following: The green fluorescent protein (GFP), derived from the Aequorea victoria jellyfish, is a highly stable reporter capable of resisting temperatures over 60°C, low pH and proteases. This statement indicates that GFP is derived from jellyfish.

Please clearly mention the software and its version, ensuring that it is distinctly different from the GFP derived from jellyfish.

Kind regards,

Karthikeyan Thiyagarajan PhD

Academic Editor, PLOS ONE.

**********

[NOTE: If reviewer comments were submitted as an attachment file, they will be attached to this email and accessible via the submission site. Please log into your account, locate the manuscript record, and check for the action link "View Attachments". If this link does not appear, there are no attachment files.]

To ensure your figures meet our technical requirements, please review our figure guidelines: https://journals.plos.org/plosone/s/figures

You may also use PLOS’s free figure tool, NAAS, to help you prepare publication quality figures: https://journals.plos.org/plosone/s/figures#loc-tools-for-figure-preparation.

NAAS will assess whether your figures meet our technical requirements by comparing each figure against our figure specifications.

# Author comment: Author response to Decision Letter 3

**DOI:** 10.1371/journal.pone.0355715.r006

20 Jul 2026

Dear reviewers,

We thank the reviewers and editor for their thorough evaluation of our manuscript and for their constructive comments. These suggestions have further improved the clarity, structure, and focus of the study. All comments have been carefully considered and addressed, as detailed below:

Comment Response

1. I suggest to do some minor corrections:

Transformant UOMSTC1R2 is mentioned in two ways: UOM STC1R2 and UOMSTC1R2 Thank you for your comment. The transformant name has now been corrected in the text, adding the space for consistency.

2. Jellyfish v.2.3.1, and you have also mentioned the following: The estimated genome size for D. tanaceti isolates was 40.7 Mbp using Jellyfish. Here 'Jellyfish' means the software, doesn't it? So, please mention the software with an appropriate version throughout the manuscript Thank you very much for the comment. The version of Jellyfish (software) has been added throughout the text.

3. in the introduction you have mentioned the following: The green fluorescent protein (GFP), derived from the Aequorea victoria jellyfish, is a highly stable reporter capable of resisting temperatures over 60°C, low pH and proteases. This statement indicates that GFP is derived from jellyfish. Thank you for the comment. The wording in the introduction has been changed accordingly to avoid confusion.

We believe that these revisions have significantly improved the clarity, focus, and scientific rigour of the manuscript. We thank the reviewers again for dedicating their valuable time and detailed feedback, and hope that the revised version meets the expectations for publication.

Kind regards,

Violeta Carrillo

Attachment Submitted filename: Response_to_reviewers_auresp_3.docx

# Editor report: Decision Letter 3

**DOI:** 10.1371/journal.pone.0355715.r007

**Authors:** Karthikeyan Thiyagarajan

26 Jul 2026

Agrobacterium tumefaciens-mediated transformation and T-DNA insertion characterisation of Didymella tanaceti and Stagonosporopsis tanaceti for infection visualisation in pyrethrum

PONE-D-25-58448R3

Dear Dr. Portillo,

We’re pleased to inform you that your manuscript has been judged scientifically suitable for publication and will be formally accepted for publication once it meets all outstanding technical requirements.

Within one week, you’ll receive an e-mail detailing the required amendments. When these have been addressed, you’ll receive a formal acceptance letter and your manuscript will be scheduled for publication.

An invoice will be generated when your article is formally accepted. Please note, if your institution has a publishing partnership with PLOS and your article meets the relevant criteria, all or part of your publication costs will be covered. Please make sure your user information is up-to-date by logging into Editorial Manager at Editorial Manager® and clicking the ‘Update My Information' link at the top of the page. For questions related to billing, please contact billing support.

If your institution or institutions have a press office, please notify them about your upcoming paper to help maximize its impact. If they’ll be preparing press materials, please inform our press team as soon as possible -- no later than 48 hours after receiving the formal acceptance. Your manuscript will remain under strict press embargo until 2 pm Eastern Time on the date of publication. For more information, please contact onepress@plos.org.

Kind regards,

Karthikeyan Thiyagarajan, PhD

Academic Editor

PLOS One

Additional Editor Comments:

Dear Authors,

After careful scientific evaluations with peer reviews, I am pleased to confirm the manuscript entitled "Agrobacterium tumefaciens-mediated transformation and T-DNA insertion

characterisation of Didymella tanaceti and Stagonosporopsis tanaceti for infection visualisation in pyrethrum" has been accepted for publication in PLOS ONE.

Kind regards,

Karthikeyan Thiyagarajan PhD

Academic Editor, PLOS ONE.

Reviewers' comments:

# Editor report: Acceptance letter

**DOI:** 10.1371/journal.pone.0355715.r008

**Authors:** Karthikeyan Thiyagarajan

PONE-D-25-58448R3

PLOS One

Dear Dr. Carrillo Portillo,

I'm pleased to inform you that your manuscript has been deemed suitable for publication in PLOS One. Congratulations! Your manuscript is now being handed over to our production team.

At this stage, our production department will prepare your paper for publication. This includes ensuring the following:

* All references, tables, and figures are properly cited

* All relevant supporting information is included in the manuscript submission,

* There are no issues that prevent the paper from being properly typeset

You will receive further instructions from the production team, including instructions on how to review your proof when it is ready. Please keep in mind that we are working through a large volume of accepted articles, so please give us a few days to review your paper and let you know the next and final steps.

Lastly, if your institution or institutions have a press office, please let them know about your upcoming paper now to help maximize its impact. If they'll be preparing press materials, please inform our press team within the next 48 hours. Your manuscript will remain under strict press embargo until 2 pm Eastern Time on the date of publication. For more information, please contact onepress@plos.org.

You will receive an invoice from PLOS for your publication fee after your manuscript has reached the completed accept phase. If you receive an email requesting payment before acceptance or for any other service, this may be a phishing scheme. Learn how to identify phishing emails and protect your accounts at https://explore.plos.org/phishing.

If we can help with anything else, please email us at customercare@plos.org.

Thank you for submitting your work to PLOS ONE and supporting open access.

Kind regards,

PLOS ONE Editorial Office Staff

on behalf of

Dr. Karthikeyan Thiyagarajan

Academic Editor

PLOS One
